# Intersections of Big Data and IoT in Academic Publications: A Topic Modeling Approach

**DOI:** 10.3390/s25030906

**Published:** 2025-02-02

**Authors:** Diana-Andreea Căuniac, Andreea-Alexandra Cîrnaru, Simona-Vasilica Oprea, Adela Bâra

**Affiliations:** 1 Department of Economic Informatics and Cybernetics, Bucharest University of Economic Studies, No. 6 Piaţa Romană, 010374 Bucharest, Romania; diana.cauniac@csie.ase.ro (D.-A.C.); alexandra.cirnaru@csie.ase.ro (A.-A.C.); bara.adela@ie.ase.ro (A.B.); 2 Doctoral School of Economic Informatics, Bucharest University of Economic Studies, 010374 Bucharest, Romania

**Keywords:** IoT, big data, Latent Dirichlet Allocation (LDA), Natural Language Processing (NLP), network, data

## Abstract

As vast amounts of data are generated from various sources such as social media, sensors and online transactions, the analysis of Big Data offers organizations the ability to derive insights and make informed decisions. Simultaneously, IoT connects physical devices, enabling real-time data collection and exchange that transforms interactions within smart homes, cities and industries. The intersection of these fields is essential, leading to innovations such as predictive maintenance, real-time traffic management and personalized solutions. Utilizing a dataset of 8159 publications sourced from the Web of Science database, our research employs Natural Language Processing (NLP) techniques and selective human validation to analyze abstracts, titles, keywords and other useful information, uncovering key themes and trends in both Big Data and IoT research. Six topics are extracted using Latent Dirichlet Allocation. In Topic 1, words like “system” and “energy” are among the most frequent, signaling that Topic 1 revolves around *data systems and IoT technologies*, likely in the context of smart systems and energy-related applications. Topic 2 focuses on the *application of technologies*, as indicated by terms such as “technologies”, “industry” and “research”. It deals with how IoT and related technologies are transforming various industries. Topic 3 emphasizes terms like learning and research, indicating a focus on *machine learning and IoT applications*. It is oriented toward research involving new methods and models in the IoT domain related to learning algorithms. Topic 4 highlights terms such as smart, suggesting a focus on *smart technologies and systems*. Topic 5 touches upon the role of digital chains and supply systems, suggesting an industrial focus on *digital transformation*. Topic 6 focuses on technical aspects such as *modeling, system performance and prediction algorithms*. It delves into the efficiency of IoT networks with terms like “accuracy”, “power” and “performance” standing out.

## 1. Introduction

The continuous flow of data from various sources, including social media interactions, embedded sensors, and online transactions, represents what is now referred to as Big Data. The scale of these data presents valuable opportunities for deeper insights, enabling businesses and organizations to make data-driven decisions. Meanwhile, the Internet of Things (IoT) connects physical devices to the internet, allowing them to gather and transmit data in real time. It is reshaping interactions with technology, influencing everything from smart home automation and urban infrastructure to industrial processes and technological solutions [1].

The convergence of Big Data and IoT is driving advancements in areas such as predictive maintenance in manufacturing, intelligent traffic systems in urban environments and personalized solutions (for instance, in healthcare). As IoT devices generate vast quantities of data, Big Data analytics has an important role in processing and interpreting this information, revealing patterns and insights that would otherwise remain undetected. This integration is accelerating progress in fields such as autonomous transportation, energy optimization and industrial operations. As a result, research into Big Data and IoT remains highly relevant for the next generation of intelligent systems and digital services [2,3].

**Figure 1:**
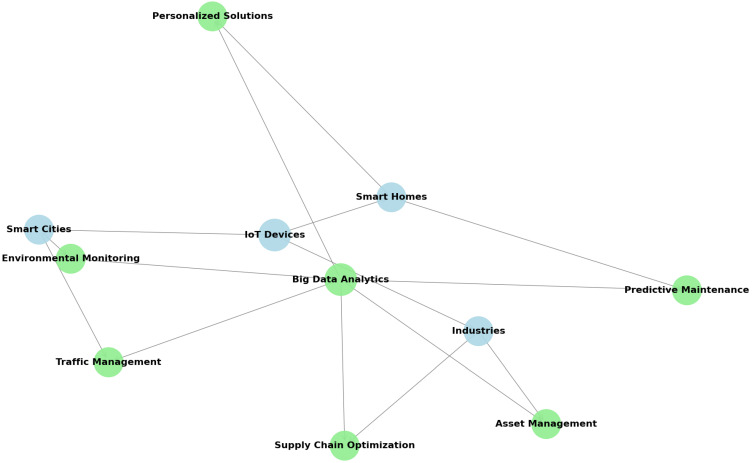
Synergy between Big Data and IoT.

The interaction between Big Data and IoT is an essential driver of transformative innovations across smart homes, smart cities and industries (the blue nodes in Figure 1 connected with IoT devices). By using IoT’s capability to collect and transmit data alongside Big Data’s analytical power, this integration facilitates real-time decision making, personalized solutions and better performance.

In smart homes, IoT-enabled devices such as thermostats, lighting systems and voice assistants collect data on user behavior and preferences. Big Data analytics processes this information to deliver personalized solutions, such as optimizing energy consumption based on daily routines. Additionally, predictive maintenance is enhanced by IoT appliances that transmit performance data to cloud-based platforms, enabling proactive maintenance scheduling and minimizing downtime.

In smart cities, IoT and Big Data are integral to optimizing city infrastructure. Traffic management systems rely on IoT sensors and GPS-enabled devices to monitor congestion and traffic flow. Then, Big Data applications optimize traffic signals, manage vehicle rerouting and enhance mobility. Environmental monitoring systems also benefit from this synergy, using IoT to track air quality, noise pollution and water conditions, while Big Data analytics identifies pollution hotspots and informs mitigation strategies.

Industries are likewise undergoing significant changes due to the combined impact of Big Data and IoT. Predictive maintenance and asset management benefit from IoT sensors that track equipment performance with Big Data processing these insights to anticipate and prevent costly failures. Supply chain management is also enhanced, as the real-time IoT tracking of shipments and inventory enables more accurate logistics planning and demand forecasting. Moreover, industrial processes become more efficient with IoT systems monitoring production flows and workforce productivity, while Big Data analytics identifies inefficiencies and improves operations.

The synergy between Big Data and IoT is graphically represented in Figure 1. The blue nodes refer to the IoT devices at the smart cities, smart homes and industry level, whereas the green nodes refer to the Big Data analytics for these entities: cities, homes, industries.

With thousands of publications released annually, understanding the core trends, influential researchers and the direction of academic inquiry becomes increasingly challenging. Our analysis offers a structured approach to navigating this vast body of knowledge, helping to identify key themes, trace the development of specific research areas and uncover the collaborative networks that drive innovation in the field.

Traditional methods of analyzing research output, such as literature reviews, while valuable, often fail to capture content-based connections between studies. This is where the application of NLP provides advantages. By adopting NLP techniques, it becomes possible to process and analyze larger text data: the abstracts, keywords and titles of academic papers, thus uncovering semantic patterns that are not immediately visible through more conventional numerical metrics such as citation counts. NLP allows for the extraction of thematic clusters and the identification of evolving research trends [4,5].

Through NLP, our research aims to reveal the dominant topics within Big Data and IoT research and also how these topics have developed over time. Moreover, it seeks to highlight the researchers and institutions that are at the forefront of innovation in these areas. By analyzing the language and content of the literature, this approach offers a nuanced perspective of the research landscape, identifying emerging trends and guiding future scholarly efforts. Ultimately, this contributes to a clearer understanding of the current state of research in Big Data and IoT and its potential future directions.

The objectives of our research focus on providing a clear understanding of the research landscape in the fields of Big Data and IoT. Through a detailed analysis, our research aims to accomplish several goals that contribute to understanding how research in these areas has evolved and where it is heading.

- First, our research seeks to identify key trends within the literature by analyzing abstracts, keywords, and titles from a large corpus of publications. This includes detecting frequently explored topics and themes as well as tracking the distribution of keywords over time.
- The research also focuses on six primary topics within the Big Data and IoT fields. These topics have been analyzed to understand how they have evolved, showing how some areas of research have gained importance while others have declined or remained steady.
- Another goal is to examine collaboration patterns between authors, institutions, and countries. By mapping co-authorship, the research identifies contributors and institutions that play a central role in Big Data and IoT research, illustrating how knowledge and innovation are shared across regions.
- Additionally, our research examines the co-occurrence of keywords in the literature to identify clusters of related research areas and show how these areas are connected. This helps to demonstrate the relationships between various topics and the interdisciplinary nature of research in Big Data and IoT. In the end, this research provides an overview of the academic landscape in these fields, helping to guide future research, foster collaboration and identify emerging areas.

The *motivation* behind our research stems from the need to navigate and understand the rapidly expanding body of knowledge in the fields of Big Data and IoT given their transformative potential across industries and society. Exploring the interdisciplinary nature of Big Data and IoT, the research emphasizes the interconnectedness of these fields with other domains, such as energy management, industrial optimization and urban development. This interdisciplinary perspective is essential for driving holistic solutions to complex challenges. Overall, our motivation is to advance understanding, foster innovation and provide actionable insights that can guide the future of research and application in Big Data and IoT.

While prior reviews on Big Data and IoT exist, our research sets itself apart by leveraging Natural Language Processing (NLP) techniques and topic modeling, such as Latent Dirichlet Allocation (LDA), to analyze a large dataset of 8159 publications. Our main motivation was to extract recent and relevant information from these publications on timely concepts such as Big Data and IoT. This data-driven approach uncovers patterns and semantic connections that traditional literature reviews often fail to identify. By extracting and analyzing six distinct thematic topics, our research provides a structured understanding of research trends, offering insights into these fields. Additionally, our research emphasizes the evolution of Big Data and IoT over time, showing how specific areas of study have grown or shifted in emphasis. It highlights the interdisciplinary nature of the fields, exploring connections between smart systems, energy applications, industrial transformations and technical aspects such as prediction algorithms and system performance. Our research also maps collaboration patterns among researchers, institutions and countries, offering valuable insights into the research ecosystem. Moreover, it identifies emerging technologies, such as edge computing, blockchain and AI, as key drivers of innovation in these fields.

Our research makes several significant contributions to the understanding of Big Data and IoT:

- First, it identifies six primary thematic topics using Latent Dirichlet Allocation (LDA) modeling, revealing key areas such as data systems and IoT technologies in smart systems and energy applications, IoT applications across industries, machine learning and IoT methods, smart technologies, industrial transformations and technical aspects like system performance and prediction algorithms.
- It also tracks the evolution of research themes over time, highlighting how specific areas, such as energy applications or industrial IoT, have grown in prominence or shifted in focus. Furthermore, the research examines keyword co-occurrence to uncover relationships between different research areas, showcasing the interdisciplinary nature of Big Data and IoT. This analysis identifies clusters of related topics, providing insights into the connections between diverse areas of study.
- A notable contribution is the identification of emerging technologies, including edge computing, blockchain, and AI, which are driving innovation in Big Data and IoT. The study sheds light on how these technologies contribute to smart systems, predictive maintenance, industrial optimization, and other transformative applications.
- By uncovering semantic patterns and connections that are often missed in traditional literature reviews, it offers a data-driven approach to understanding research trends. This approach enhances the ability to navigate and interpret the rapidly expanding body of knowledge in these fields. It emphasizes the interdisciplinary connections between Big Data and IoT, particularly in areas such as smart systems, energy management, industrial operations, and urban development.

The organization of this paper follows a systematic structure. It begins with an introduction (Section 1) that establishes the synergy between Big Data and IoT, emphasizing their transformative potential across smart homes, cities and industries. This section highlights the research gap, detailing the absence of integrated bibliometric and topic modeling analyses in existing literature. It further outlines the objectives and motivation of the study, setting the stage for the subsequent sections. Section 2 delves into the literature review, which provides an extensive examination of prior research in the fields of Big Data and IoT. This includes bibliometric studies focused on their applications in healthcare, agriculture and industrial IoT. The methodology (Section 3) describes the research process in detail, beginning with data collection from the Web of Science database, which resulted in a dataset of 8159 publications. It outlines the data preprocessing steps, including cleaning, tokenization and lemmatization, which is followed by sentiment analysis techniques. The use of Latent Dirichlet Allocation (LDA) for topic modeling is explained, along with visualization tools like pyLDAvis, which are employed to analyze the relationships between topics and uncover hidden thematic structures.

In the results (Section 4), the paper presents a detailed analysis of publication trends, such as the dominance of original research articles and the leading journals in the field. The outcomes of topic modeling are presented, revealing six key themes, including smart systems, IoT applications and advancements in Big Data technologies. The conclusions (Section 5) synthesize the key findings. Additionally, the potential for exploring emerging technologies like blockchain and edge computing is discussed.

## 2. Literature Review

### 2.1. Big Data and IoT

The synergy between IoT and Big Data is foundational to the advancement of smart systems across various domains. IoT generates vast amounts of real-time data through its interconnected devices—sensors, wearables, smart meters, and other IoT technologies—across industries such as healthcare, transportation, smart cities, and manufacturing. In essence, IoT serves as the data source, continuously producing information that needs to be stored, processed, and analyzed efficiently. Big Data technologies, such as Hadoop, Spark, and cloud computing, provide the infrastructure and analytical models to handle this volume, velocity, and variety of data. This synergy allows organizations to achieve smarter, more efficient operations, improve customer experiences, and innovate with advanced features like real-time analytics, predictive maintenance, and automation.

The article “Latest advancements and prospects in the next-generation of Internet of Things technologies” contributes to this understanding by emphasizing the critical role of Big Data analytics in IoT ecosystems [6]. The authors highlight how Big Data can be leveraged to extract valuable insights from the extensive data generated by IoT devices. By analyzing patterns, trends, and correlations in the IoT-generated data, Big Data technologies enhance decision-making processes, support predictive models, and enable the creation of personalized services tailored to individual or organizational needs. The paper underlines that this combination not only optimizes IoT system performance but also fosters the development of more intelligent and autonomous systems.

Moreover, the article delves into the challenges faced by IoT systems, particularly concerning data management, security, and privacy. Big Data tools offer solutions to these challenges by providing scalable data storage, real-time processing capabilities, and advanced analytics that improve the efficiency of IoT systems. The paper underscores that the next generation of IoT technologies will increasingly rely on the synergy between IoT and Big Data to overcome existing limitations and unlock new potential in smart applications. Through this synergistic relationship, the capabilities of IoT can be maximized, ensuring that the massive flow of data is harnessed effectively to drive intelligent outcomes.

The rapid advancement of Big Data and the Internet of Things (IoT) has generated extensive academic interest, which is reflected in a significant number of publications. Recent bibliometric analyses offer a systematic view of the research landscape in these fields, identifying key themes, methodologies, and research gaps. For instance, analyses conducted by MDPI and IEEE Xplore have highlighted several recurring themes, including real-time data processing, smart city applications and healthcare IoT implementations [7].

Bibliometric studies reveal that collaboration in Big Data and IoT research has expanded significantly over recent years, with co-authorship networks centered around countries with substantial technological infrastructures, such as the United States and China. Research clusters often focus on applications in smart cities, healthcare and industrial IoT, where real-time analytics and data integration are paramount challenges.

Tools like VOSviewer and SciMAT are frequently used to map author collaboration, topic co-occurrences, and research productivity, helping to visualize the field’s evolution and highlight prolific authors and institutions. For example, Özköse, in his study “Bibliometric Analysis and Scientific Mapping of IoT”, used SciMAT as a tool for understanding the existing landscape and potential growth in IoT research [8].

The article “Exploring Machine Learning: A Scientometrics Approach Using Bibliometrix and VOSviewer” analyzes the machine learning research landscape through bibliometric methods. The authors used VOSviewer and Bibliometrix to visualize trends in publications, identify influential authors, and uncover key research themes. They present some significant contributions in the field and illustrate collaboration networks among researchers, as well as the increasing relevance of machine learning across various disciplines, suggesting future research directions [9].

Goranin et al. in their article “A Bibliometric Review of Intrusion Detection Research in IoT” offer a comprehensive look at the evolution of intrusion detection systems (IDSs) in IoT networks [10]. The methodology in the article involved a bibliometric analysis to examine intrusion detection research trends in IoT. The authors collected data from the Web of Science database, focusing on research published from 2017 to 2023. They used statistical and network analysis tools to assess publication volume, keyword co-occurrences, citation patterns, and collaboration networks among researchers and institutions. Visual mapping software, such as VOSviewer, helped visualize trends and emerging areas. By leveraging tools like VOSviewer, the authors identify high-impact research areas such as machine learning applications in IDSs, challenges around data security and privacy, and the growing importance of real-time detection systems in IoT. The review suggests research directions, including enhancing algorithmic efficiency and increasing international collaboration to address IoT security challenges. This approach provided insight into high-impact topics, key contributors, and collaborative efforts in IoT intrusion detection research [10].

Parlina et al. examined trends in Big Data research from 2009 to 2018, focusing on topics, collaboration networks, and keyword analysis using data from Scopus [11]. Researchers used co-word analysis and VOSviewer to map topics, identifying significant themes like machine learning, data analytics, and management. They found Big Data research increasingly intersects with fields like healthcare, finance, and social sciences, concluding that its interdisciplinarity reflects growing practical and theoretical complexity.

The authors Abdullahi et al. conducted a bibliometric analysis to assess the evolution of Internet of Things (IoT) applications in smart agriculture [12]. They utilized data from the Scopus database covering publications from 2012 to 2022. They employed co-citation analysis and keyword co-occurrence methods, leveraging VOSviewer software to visualize trends, collaborations, and thematic clusters in the research landscape. The analysis revealed a significant increase in publications related to IoT applications in agriculture, particularly from 2017 onwards. Key themes identified include precision agriculture, remote sensing, and smart farming technologies. The study highlights strong collaborations among institutions with notable contributions from regions such as Europe and North America. The authors suggest that future research should focus on developing more integrated IoT systems to address challenges in smart agriculture, emphasizing sustainability and resource efficiency.

Emerging trends in the literature focus on specific technological and ethical challenges. For instance, while advancements in IoT hardware and real-time processing capabilities are regularly highlighted, studies also emphasize the ethical implications surrounding data privacy and security. Notably, the integration of Big Data analytics with IoT technologies introduces new layers of complexity in managing vast data volumes, particularly when sensitive information is involved in domains like healthcare and urban planning [13,14].

Despite significant advances, bibliometric analyses identify critical gaps that future research could address. Areas requiring deeper exploration include enhanced frameworks for data interoperability across IoT platforms and advanced machine learning algorithms to improve predictive capabilities within Big Data environments [15].

Additionally, there is a noted need for studies focusing on scalable architectures that support the growing data influx in IoT systems. As the field matures, further bibliometric studies may continue to map the evolving intersections of Big Data and IoT, providing actionable insights for both academia and industry.

Another prominent field is smart city development, where IoT and Big Data are essential for managing urban resources and infrastructure. In their bibliometric analysis, Alaeddini et al. identified primary themes around energy management, traffic optimization, and environmental monitoring. The authors used cluster analysis to show how Big Data technologies enable smart city frameworks to make data-driven decisions. They suggest that future studies focus on integrating machine learning with IoT in real time to address the complex demands of urban populations. These technologies not only help in monitoring but also provide predictive insights that can aid in urban planning [16].

The article “An Edge-Fog-Cloud computing architecture for IoT and smart metering data” introduces a comprehensive computing architecture that integrates Edge, Fog, and Cloud computing to process data from IoT devices and smart metering systems [17]. The authors propose a framework that distributes data processing tasks across these three layers to enhance efficiency and reduce latency. By allocating specific tasks to each layer—Edge, Fog, and Cloud—the architecture aims to optimize resource utilization and improve the overall performance of IoT applications.

The paper emphasizes the importance of this layered approach in managing the diverse and voluminous data generated by IoT devices and smart meters. The Edge layer handles immediate data processing needs, the Fog layer manages intermediate processing and storage, and the Cloud layer provides extensive computational resources for complex analytics and long-term data storage. This hierarchical structure ensures that data are processed at the most appropriate level, balancing the trade-offs between speed, resource consumption, and computational power.

In “A bibliometric analysis of IoT applications in logistics and supply chain management”, the authors delve into the integration of IoT within the logistics and supply chain management sector [18]. This study provides a comprehensive bibliometric overview, exploring how IoT technologies optimize processes in logistics and supply chains, particularly focusing on Big Data analytics and IoT-driven innovation. The paper emphasizes the potential of IoT to enhance the food supply chain, highlighting how data from IoT devices can be used for better resource management and more efficient operations. Through citation analysis and trends, the study captures the rapid evolution of these technologies, offering insights into the growing importance of IoT in transforming supply chain dynamics.

The article “Education big data and learning analytics: a bibliometric analysis” analyzes trends in the intersection of Big Data and education, specifically focusing on learning analytics [19]. It presents a thorough examination of how Big Data are being utilized in educational settings to track learning patterns and improve student outcomes. The paper not only reviews the existing literature but also provides a forward-looking perspective, offering recommendations for the future of learning analytics. The authors point out the potential for Big Data to revolutionize personalized learning and enhance pedagogical methods, encouraging further research to refine educational practices and technologies.

Lars Lundberg, in his study, offers an in-depth exploration of the ever-evolving Big Data field, aiming at identifying key research trends and future directions in a rapidly growing domain [20]. He used bibliometric methods to analyze the vast body of literature related to Big Data, uncovering critical themes such as data processing, storage, and privacy concerns. The author also touched on emerging areas like artificial intelligence and machine learning, emphasizing the interdisciplinary nature of Big Data research. This paper is pivotal for researchers seeking to understand the broad scope of Big Data and its implications for various sectors.

The paper “A Bibliometric Analysis of Research on Big Data and Its Potential to Value Creation and Capture” focuses on the scientific landscape of Big Data specifically in terms of its potential for value creation and capture [21]. The study applies bibliometric techniques to assess how Big Data can drive economic growth and business value. By reviewing academic literature on the subject, the authors outline the evolving role of Big Data in shaping business strategies, operational efficiencies, and innovations across industries. This analysis is particularly insightful for understanding the relationship between Big Data and organizational value creation, offering valuable insights for both scholars and industry professionals.

In “Visualizing the Landscape of Home IoT Research: A Bibliometric Analysis”, the authors provide a detailed overview of the research landscape surrounding home IoT. Bibliometric methods were used to map the key trends, challenges, and future directions in the domain of smart home technologies. They focus on the various IoT devices and systems used in homes, from security solutions to energy management systems, and explore how these technologies are shaping modern living environments. The study identifies gaps in research, particularly in areas such as user privacy, device interoperability, and the integration of AI with IoT in the home context [22].

Wang et al., in the reference [23], examine the role of edge computing in the IoT ecosystem, focusing on how it facilitates the processing of Big Data generated by IoT devices. This paper provides valuable insights into the convergence of edge computing and IoT, detailing how edge computing can enhance data processing speed, reduce latency, and improve the efficiency of IoT networks. Through a bibliometric analysis, the study highlights the rapid advancements in edge computing technologies and the growing interest in this field within IoT research.

Each of these studies uses bibliometric methods to analyze vast domains of research, offering a critical examination of trends, emerging topics, and gaps in their respective fields. From logistics and education to IoT and Big Data, these articles highlight the interconnectedness of technological advances and their potential to shape industries in profound ways.

Collectively, these studies point to several challenges in Big Data and IoT integration, notably in data security, interoperability, and the need for scalable infrastructure. As IoT ecosystems grow, managing the sheer volume and complexity of data requires advancements in real-time analytics and machine learning. Ge et al. present in their article titled “Big Data in Internet of Things: A Survey” a comprehensive overview of how Big Data are utilized within IoT ecosystems. It outlines the unique challenges presented by the massive volumes of data generated by IoT devices, including data storage, processing and analysis [24]. The authors explore various architectures and frameworks designed to facilitate effective Big Data management in IoT contexts. Additionally, the survey highlights potential applications, future trends and the need for advanced analytical techniques and security measures.

Each domain presents unique requirements, whether in patient privacy in healthcare or real-time reliability in industrial settings [25]. Future research may thus benefit from cross-disciplinary approaches that address these foundational challenges while continuing to advance the specific needs of each domain.

In an extensive bibliometric analysis on Big Data and IoT in smart infrastructure, Guo et al. used data from Web of Science to map publication trends, highlighting rapid growth in areas like real-time monitoring, data visualization, and smart grids [26]. Their study identified clusters around infrastructure resilience, energy efficiency, and smart transportation, underscoring the role of Big Data in enhancing IoT-driven urban management. The authors also found that collaboration among industry and academia has increased, particularly in regions investing in smart city initiatives.

The adoption of IoT and Big Data in healthcare has been examined by Šajnović and her co-authors. This analysis focused on wearable technologies and predictive analytics for patient care, showing a surge in publications related to chronic disease management and real-time health monitoring. According to them, Big Data enable IoT devices to generate insights for personalized healthcare, though challenges such as data privacy and interoperability remain prominent. Their analysis reveals the importance of data governance in healthcare IoT, advocating for secure, interoperable systems to manage sensitive health data effectively [27].

IoT and Big Data have also seen significant application in agriculture, as evidenced by a bibliometric study by Misra et al. This research highlights IoT’s role in precision agriculture, where real-time data on soil conditions, crop health, and weather forecasts enable farmers to optimize resources. The authors note a growth in publications on environmental sustainability and resource conservation with keywords such as “climate adaptation” frequently appearing. They call for future research on robust analytics frameworks that can process large, diverse agricultural data to address global food security concerns [28].

In the industrial sector, IoT applications for predictive maintenance and asset management have been a focal point, as explored in a bibliometric study by Keleko et al. Their analysis reveals a rising interest in IoT applications for supply chain optimization and automated quality control. Big Data analytics allows organizations to anticipate equipment failures and optimize production through predictive maintenance, underscoring the need for robust data management strategies and the integration of advanced analytics. The authors advocate for future research to address challenges related to implementation and scalability [29].

IoT plays an important role in the educational sector as well. The article “A Comparative Study of Chinese and Foreign Research on the Internet of Things in Education: Bibliometric Analysis and Visualization” analyzes publications related to IoT in education, comparing trends between Chinese and international research. The study reveals distinct research focuses with China emphasizing smart campus implementations while foreign research tends to explore broader applications. It concludes by suggesting areas for future research and collaboration to enhance the integration of IoT in educational contexts [30].

### 2.2. Smart Industries

The intersection of Big Data and IoT technologies continues to attract significant scholarly interest across diverse domains, including agriculture, industrial management, and business. A review of recent bibliometric analyses highlights evolving research trends and emerging thematic clusters within these fields [31].

For instance, IoT’s role in smart agriculture has been extensively analyzed by Liang and Shah, who mapped out core themes such as precision agriculture, climate change adaptation, and smart irrigation. The study emphasizes that IoT adoption in agriculture often leverages wireless sensor networks and machine learning to optimize water usage and soil management, ultimately addressing food security challenges. The authors identified a dramatic increase in IoT-related publications in agriculture, with topics like machine learning and smart irrigation showing high growth rates, indicating both scholarly and practical interest in enhancing agricultural productivity [32].

Research shows the growing role of IoT in agriculture, emphasizing its potential to address food security and environmental challenges by integrating advanced data-driven technologies such as blockchain, UAVs, and IoT networks to monitor soil, crops, and resource use efficiently. Studies on Industry 4.0 technologies in agriculture reveal that IoT and Big Data analytics are crucial for managing vast agricultural data volumes, helping to optimize crop yields and reduce environmental impact [33].

Similarly, a bibliometric study by researchers in the field of industrial IoT (IIoT) explores IoT applications in business management and operational efficiency. According to recent analyses, IIoT’s adoption within manufacturing has been pivotal with applications in automated production, industrial safety, and smart maintenance. Such research has frequently targeted the enhancement of industrial processes through IoT-driven automation and asset management, illustrating the operational impact of IoT beyond traditional IT applications. Key findings suggest a need for further research on interoperability and cybersecurity within industrial IoT systems [34,35].

On the business side, IoT has been instrumental in creating new business models and customer engagement strategies. Bibliometric analyses show that scholars are increasingly interested in IoT as a driver of innovation from smart consumer products to service personalization. Researchers have mapped trends in IoT’s role in customer profiling, secure transaction processing, and digital wallets, emphasizing IoT’s transformative potential in areas such as finance and consumer electronics [36].

A comprehensive bibliometric analysis focusing on IoT in environmental sustainability reveals a strong emphasis on IoT applications in resource conservation and climate monitoring. IoT technologies are seen as vital for enhancing sustainability practices through energy management, water conservation, and real-time environmental monitoring. This area remains a dynamic research field with publications rapidly increasing as governments and industries invest in IoT for environmental resilience [37]. These studies collectively underscore IoT’s versatility across various domains. They also highlight challenges related to data integration, privacy, and system interoperability that continue to shape research directions in Big Data and IoT technologies.

The article “Anomaly Detection with Machine Learning Algorithms and Big Data in Electricity Consumption” explores the application of machine learning (ML) techniques to identify anomalies in electricity usage data [38]. In this study, the authors analyze large datasets of readings provided by smart meters installed in a trial study in Ireland. They apply a hybrid approach that combines various machine learning algorithms to detect anomalies in electricity consumption patterns. The research aims to enhance the accuracy and efficiency of anomaly detection systems, which is crucial for identifying fraudulent activities, equipment malfunctions, or other irregularities in electricity usage.

By leveraging Big Data analytics, the study addresses the challenges associated with processing and analyzing large volumes of electricity consumption data. The integration of machine learning algorithms with Big Data enables the development of more robust and scalable anomaly detection systems, leading to improved energy management and reduced operational costs.

In a bibliometric study focused on IoT and Big Data in healthcare, Dian, Vahidnia and Rahmati identified increasing interest in wearable devices, predictive analytics, and patient monitoring. Their analysis revealed clusters of research around chronic disease management and real-time data monitoring systems. This growth is driven by the need for continuous patient data to support preventative healthcare and timely intervention, where Big Data analytics help process the extensive data streams generated by IoT devices. This study highlights key challenges, including data security and interoperability among healthcare IoT devices, as pressing issues for future research [39].

IoT and Big Data applications in agriculture and environmental monitoring have gained attention, as shown in a 2023 bibliometric analysis by Pachouri et al. This study identifies IoT’s use in precision agriculture for resource conservation, especially in water management and soil monitoring. Big Data analytics are essential for making data-driven decisions, where IoT sensors track crop health and environmental conditions in real time. The authors found that as climate change concerns grow, so does research in this area with an emphasis on using IoT for sustainable agriculture practices [40,41].

### 2.3. Smart Homes

The convergence of IoT, Big Data, AI and sustainable technologies has significantly impacted the development of smart homes and cities. Smart cities aim to enhance urban efficiency and sustainability, employing IoT and wireless sensor networks for resource optimization and infrastructure management [42]. In particular, urban water management strategies, leveraging smart technologies, address critical issues like water conservation and distribution [43]. These advancements contribute to meeting global sustainability goals and improving city resilience.

Smart homes are transforming residential spaces by integrating intelligent systems tailored for specific user needs, particularly older adults. Bibliometric analyses reveal a focus on assistive technologies for health monitoring, fall detection and personalized care [44,45]. In reference [46], the authors map a decade of research on smart homes for the elderly with a specific focus on studies indexed in Web of Science. They use CiteSpace for scientometric analysis, emphasizing co-citation patterns and keyword clustering. This research takes a longitudinal approach, analyzing how the research on smart homes for elderly individuals has evolved over the past decade. The authors identify shifts in research focus, from basic technology implementation to the integration of health monitoring, safety and comfort features. The use of CiteSpace is integral in visualizing these shifts and providing a comprehensive overview of the trends in smart homes for the elderly.

Innovative designs in smart buildings emphasize safety and comfort, addressing risks for vulnerable populations, as depicted in [47]. This research used a systematic and bibliometric analysis to explore the development of smart buildings that reduce indoor risks for safety and health, particularly for the elderly. The authors combine citation analysis with thematic clustering, using tools such as VOSviewer and CiteSpace to map out the evolution of smart building technologies. They analyze the intersection of smart technologies, building safety and elderly health. This combination of bibliometrics and systematic review helps the authors highlight emerging trends in smart building design and identify gaps in research, especially related to safety risks and environmental factors affecting elderly residents. Furthermore, IoT-driven solutions are revolutionizing indoor air quality monitoring, showcasing the potential of real-time data collection for environmental health [46].

Security and privacy remain prominent concerns, as highlighted in research exploring IoT frameworks for smart homes. Effective strategies to mitigate risks are necessary to foster user trust and system adoption [48]. In this research, the authors discuss various security protocols and solutions that have been proposed in the literature to safeguard smart home systems. Their ideas contribute to the growing body of knowledge on cybersecurity in IoT-enabled environments, emphasizing the importance of securing personal data and preventing unauthorized access. Moreover, systematic reviews of trends emphasize the importance of interdisciplinary research, suggesting that future advancements will hinge on collaborative efforts between technologists and urban planners [49,50].

### 2.4. Smart Cities

Smart cities are rapidly evolving into key areas of interdisciplinary research that span across domains like sustainability, technology, governance, urban planning and education. Bibliometric analyses have become an essential tool to systematically map the growing body of knowledge in smart city research. In the past few years, a number of studies have employed various bibliometric methods to provide insights into trends, collaborations and emerging areas of study. For instance, the research by Guo et al. provides a broad overview of smart city research through an extensive bibliometric analysis using VOSviewer and CiteSpace. This research analyzes over 4000 papers to map the co-authorship networks, identify key research clusters and examine the evolution of smart city topics. The authors focus on the identification of emerging trends over a span of two decades, providing a longitudinal perspective on the growth of the field. Their method primarily utilizes co-authorship and keyword co-occurrence to identify and visualize the intellectual landscape of smart cities. By mapping collaborative relationships and the frequency of specific terms, they offer a comprehensive view of how interdisciplinary collaborations have shaped smart city research. It serves as a foundational work for understanding the broader scope of smart city research and its global development.

In contrast, Scala et al. take a more niche approach by exploring the conceptualization of smart cities specifically in the context of education [51]. Their bibliometric study employs co-word analysis and thematic clustering techniques to map how the concept of education has been integrated into the broader smart city paradigm. The study focuses on publications from Scopus and Web of Science, employing keyword co-occurrence analysis to identify the dominant themes in smart city education. This approach allows them to visualize the evolution of educational concepts within smart cities and how they intersect with urban technologies. By employing thematic mapping, Scala et al. offer a fresh perspective, focusing on the role of education in urban environments and how it contributes to the development of smart cities. This specialized focus on education sets their study apart from the broader, more general studies in the field.

Rejeb et al. present an innovative combination of bibliometric analysis and main path analysis to trace the intellectual development of smart city research [52]. While bibliometric methods such as citation and co-citation analysis are used to map out key articles and authors, the main path analysis adds an additional layer of sophistication by identifying critical research trajectories that have shaped the evolution of the field. The main path method focuses on citation chains to reveal how foundational studies have influenced the direction of subsequent research. This dual approach of bibliometric mapping and main path analysis is particularly valuable for understanding the underlying pathways of knowledge flow in smart city research.

In a more focused area of smart city technology, Gupta et al. examine the intersection of AI and smart cities [53]. Their bibliometric analysis leverages citation analysis and keyword co-occurrence to uncover trends related to the use of AI in urban management. By employing bibliographic coupling, the study identifies the relationship between seminal papers and newer publications, providing insights into how AI is becoming integrated into smart city frameworks. Although the scope of the study is narrower in comparison to Guo et al.’s, it provides valuable insight into the increasing role of AI technologies in enhancing urban efficiency, sustainability, and overall city management [26,53]. The study’s specific focus on AI as a transformative tool for smart cities offers a perspective in the rapidly evolving field of AI applications in urban environments.

The primary goal of Hajoary et al.’s study is to map the research landscape, highlighting the most prolific researchers, institutions, and countries contributing to smart city scholarship. Additionally, it examines the thematic evolution of the field, identifies collaboration networks within the research community, and pinpoints emerging areas of focus [54]. While the study’s strength lies in its comprehensive and methodologically rigorous approach, it does have some limitations. The exclusive focus on English-language publications may exclude significant contributions in other languages, and its academic orientation does not encompass practical insights available from industry reports or patents.

Puliga et al. introduce an innovative methodological approach by combining a bibliometric analysis of scientific publications with patent analysis [55]. While many bibliometric studies focus solely on academic papers, like the article from reference [54], these authors expand the scope to include patents, offering a more holistic view of smart city innovations. By utilizing tools such as VantagePoint, they analyze both scientific publications and patent trends to uncover research and technological developments in the smart city sector. This approach is particularly valuable as it captures the practical, industry-driven aspects of smart cities alongside theoretical research. Their study emphasizes the importance of standardization and interoperability challenges in the smart city sector, shedding light on the industrial applications that stem from academic research.

A brief comparison of previous studies is provided in Table 1.

The cross-analysis of the previous studies reveals several patterns and distinctions in their objectives, methods, findings and focus areas. Most studies share a common interest in mapping research trends, highlighting interdisciplinary applications and identifying research gaps. Studies such as [7,8,10,11] focus broadly on thematic mapping in IoT and Big Data, whereas others, like [12,24,39] delve into specific sectors such as agriculture, emphasizing precision farming, food security and sustainability. Healthcare is another frequently explored area with studies like [25,27] focusing on wearable technologies and real-time patient monitoring. Industrial IoT studies, including [29,34,35], emphasize predictive maintenance and operational efficiency, demonstrating IoT’s impact beyond traditional IT applications. Ethical and technical challenges, such as privacy, data security and system interoperability, are central to studies like [13,14,15].

Methodologically, bibliometric analysis dominates, as seen in [8,10,11] and others. These studies often rely on tools like VOSviewer and SciMAT to map collaborations, topic co-occurrences, and thematic clusters. Cluster analysis, used in [16,26], identifies key themes, particularly in smart city applications and infrastructure resilience. Some studies, like [24], take a survey-based approach to provide broader overviews of Big Data and IoT ecosystems.

The findings across previous studies consistently highlight the interdisciplinary growth of IoT and Big Data research. Studies like [7,9,10,11] observe their intersection with various fields, including healthcare, agriculture and urban planning. Sector-specific insights show that IoT significantly enhances precision agriculture [12,28], patient monitoring and healthcare efficiency [27,39] and industrial automation [29,34]. Emerging trends, such as sustainability-focused IoT applications [37,40,41] and smart city innovations [26], indicate growing interest in addressing global challenges like resource conservation and urban management. However, challenges such as data scalability, integration and privacy remain persistent, as highlighted in [13,14,15,25].

While many previous studies share similarities, such as recurring themes of real-time analytics, collaboration networks and thematic exploration, they differ in their specific areas of focus. Some studies prioritize broad overviews [7,9], while others concentrate on implementation in specific sectors [12,39]. Ethical and technical challenges are central to studies like [13,14,25], whereas [34,35] emphasize operational benefits and automation. Several gaps remain across the reviewed studies. While challenges like interoperability and scalability are widely recognized, actionable frameworks to address them are less explored. Emerging areas such as IoT in education [30] and blockchain’s integration with IoT [25] are also underrepresented.

In terms of methodology, the last five studies share a common reliance on bibliometric tools like CiteSpace, VOSviewer and Scopus, which are standard for mapping research trends and visualizing networks of knowledge. However, the studies differ in the scope of their focus and the tools they combine with bibliometric analysis. The authors of [54] provide a comprehensive, longitudinal overview, while the authors of [51] focus on the role of education within the smart city paradigm. Rejeb et al. introduce the novel use of main path analysis to trace intellectual trajectories, adding depth to the bibliometric approach [52]. Gupta et al. narrow their focus to AI applications, shedding light on a specific technological aspect of smart cities [53]. Puliga et al., on the other hand, incorporate patent analysis, bridging the gap between academic research and industrial innovation, offering a more comprehensive view of how smart city concepts are applied in practice [55]. Each study contributes to the growing body of knowledge on smart cities, but they do so using different methods that reflect the diversity and complexity of the field. Hajoary et al. provide a broad mapping of the field’s development, Scala et al. explore specific educational frameworks, whereas Rejeb et al. use a methodological innovation to map the flow of ideas through the research landscape [51,52,54]. Gupta et al. bring attention to the growing importance of AI in smart city development, while Puliga et al. offer a dual analysis of academic publications and patents, presenting a more practical, industry-oriented perspective [53,55].

To the best of the authors’ knowledge, the intersection of Big Data and IoT has not yet been comprehensively examined through the combined methodologies of bibliometric analysis and topic modeling. While previous studies (as presented in this section) have separately explored advancements in Big Data and IoT and have employed bibliometric tools to analyze research trends within these fields individually, a systematic and integrated approach that investigates their intersection using advanced techniques such as topic modeling remains unexplored. This *gap* is significant because the convergence of these two domains represents a critical area of technological innovation, influencing diverse applications such as smart cities, healthcare and industrial automation.

By employing bibliometric analytics alongside topic modeling, such as Latent Dirichlet Allocation (LDA), it becomes possible to uncover not only the dominant research themes and trends but also the evolving relationships between these interconnected fields. How Big Data and IoT research areas complement each other is revealed, identifying thematic clusters at their convergence and highlighting emerging research trajectories that traditional bibliometric analyses might overlook.

## 3. Research Methodology

The dataset used in our research encompasses nine full record files, collectively containing information on 8159 publications related to Big Data and IoT research. These records have been sourced from Web of Science, a leading research database that includes high-quality, peer-reviewed publications. The timeframe covered by the dataset captures the evolution of research in these fields, providing a broad overview of key developments and trends over the years.

Each data file provides essential bibliographic details such as author names, article titles and information about the conference or journal where the research was presented or published. This allows for a complex analysis of collaboration patterns among researchers and institutions as well as an understanding of the most influential venues for disseminating knowledge in Big Data and IoT.

Additionally, the datasets contain keywords and abstracts that reveal the main themes and highlights about all areas of the research. These textual elements are particularly valuable for NLP techniques, which will be used to analyze patterns and trends in the text of the literature. By extracting key terms and recurring topics, it becomes possible to identify emerging research areas and map how certain themes have evolved.

Overall, this dataset provides a foundation for exploring the collaborative networks between authors and institutions, uncovering key trends within the literature and conducting an analysis of the content through NLP. By leveraging this dataset, our research aims to offer a detailed overview of the academic landscape in Big Data and IoT, contributing to a clearer understanding of how research in these fields is developing and where future opportunities for innovation may lie.

**Figure 2:**
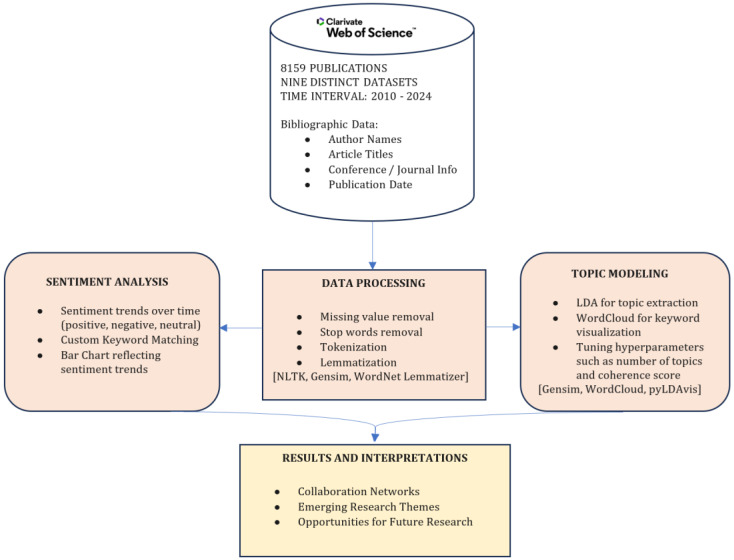
Methodology flowchart.

The research methodology is described in Figure 2. First, data are collected from the Web of Science platform, comprising 8159 publications from nine full record files within the time interval of 2010 to 2024. The results were obtained using the following search:

$$\left(\left(AF=\mathrm{big}\;\mathrm{data}*\right)˄\left(\left(AF=\mathrm{IoT}\right)˄\left(AF=\mathrm{Internet}\;\mathrm{of}\;\mathrm{Things}\right)\right)\right)\left(˄\left(DT=\mathrm{Article}\right)\right)\left(˄\left(LA=\mathrm{English}\right)\right)(˄\neg (RN\;=\mathrm{Retracted}\;\mathrm{Publication}))\left(˄\neg \left(PY<2010\right)\right)\;\;\;\;\;\;\;\;\;$$

where

AF—All Fields;
DT—Document Types;
OA—Open Access;
LA—Language;
RN—Retraction Notices;
PY—Publication Years;
*—any word following big data;
˄—AND;
¬—NOT.

Thus, the targeted publications are at the intersection of these two concepts: Big Data and IoT. Publications that focused only on Big Data or IoT were not included in the input data. Bibliographic data include essential fields such as author names, article titles, conference or journal information, and publication dates. Next, the Data Processing stage involves cleaning the data through steps like missing value removal, stop words removal, tokenization, and lemmatization, employing tools such as NLTK, Gensim and the WordNet Lemmatizer. In the Sentiment Analysis phase, sentiment trends over time are evaluated using custom keyword matching, and a bar chart is generated to visualize the sentiment distribution.

An alternative strategy for sentiment analysis in specialized fields is to create custom word lists tailored to the specific context of the research. This process involves selecting and categorizing words and phrases that are directly relevant to topics such as Big Data and IoT. Sentiment scores are then assigned to these terms based on their contextual implications. For example, in this domain, words like “innovation”, “performance”, “efficiency”, or “breakthrough” could signify positive sentiment, while terms such as “uncertainty”, “decline”, or “challenge” might indicate negative sentiment. Neutral terms might consist of technical or descriptive language that carries no inherent emotional weight. This approach is advantageous for exploring specialized or niche areas, where generic sentiment analysis tools may overlook critical nuances in terminology.

For Topic Modeling, the LDA technique is used to extract topics along with WordCloud for keyword visualization. Hyperparameters, such as the number of topics and coherence score, are tuned using tools like Gensim, WordCloud, and pyLDAvis.

Finally, the Results and Interpretations stage identifies collaboration networks, emerging research themes, and provides opportunities for future research.

In our research, NLP techniques are applied to analyze the large volume of textual data related to Big Data and IoT research. Given the vast number of abstracts, titles and keywords, these methods help extract key concepts, identify patterns and uncover important trends within literature.

The first step in the data processing involves breaking down the text into smaller units using tokenization, allowing for the identification of frequently mentioned terms. To ensure consistency and reduce redundancies, processes like stemming and lemmatization are applied, which normalize words by reducing them to their base forms.

A major part of the analysis involves topic modeling, which is a technique that helps detect underlying themes across the body of literature. By doing so, it will be possible to identify the primary areas of research within Big Data and IoT as well as track how these topics have evolved. Additionally, our research employs techniques like TF-IDF to highlight the most relevant and distinctive terms within the dataset, providing insights into specialized areas of research.

The data processing and analysis are carried out using Python, along with specialized libraries for text analysis and data manipulation, ensuring an efficient and structured exploration of the dataset. Together, these methods offer an understanding of the research landscape in Big Data and IoT. The methodological steps and parameters are summarized in Table 2.

This structured approach presents the methodology flow, detailing each step of the analysis with specific parameters. The importance of integrating validation steps, even in fully automated studies, is obvious, in order to ensure that conclusions drawn from large datasets are robust and defensible. Studies like Systematic Mapping Studies (SMSs) or similar approaches that summarize the existing literature are reliable. While our research relies on automated NLP techniques with a human-validation step, the proposed methodology has both advantages and limitations compared to manual or hybrid methods commonly used in SMS. Automated vs. manual processes pose interesting challenges. The exclusive reliance on NLP techniques ensures objectivity and scalability, allowing for the analysis of large datasets like the 8159 publications. Automation minimizes biases introduced by human decision making, particularly during source inclusion/exclusion. However, as noted, the absence of manual screening and verification can raise concerns about the reliability of the conclusions. Manual checks by multiple researchers, as seen in traditional SMSs, provide a form of triangulation that strengthens the validity of the results. To enhance the credibility of an automated NLP-driven approach, we have included a validation step to cross-check results. For instance, we compared a subset of NLP-identified themes with manual interpretations by domain experts, ensuring alignment between automated insights and human understanding. While the study leverages the power of NLP to process a vast dataset efficiently, integrating a hybrid approach (automated processing supplemented by selective manual validation) strengthens its reliability. For example, we manually reviewed a small percentage of abstracts or themes to assess the accuracy of the NLP-generated outputs.

## 4. Results

**Figure 3:**
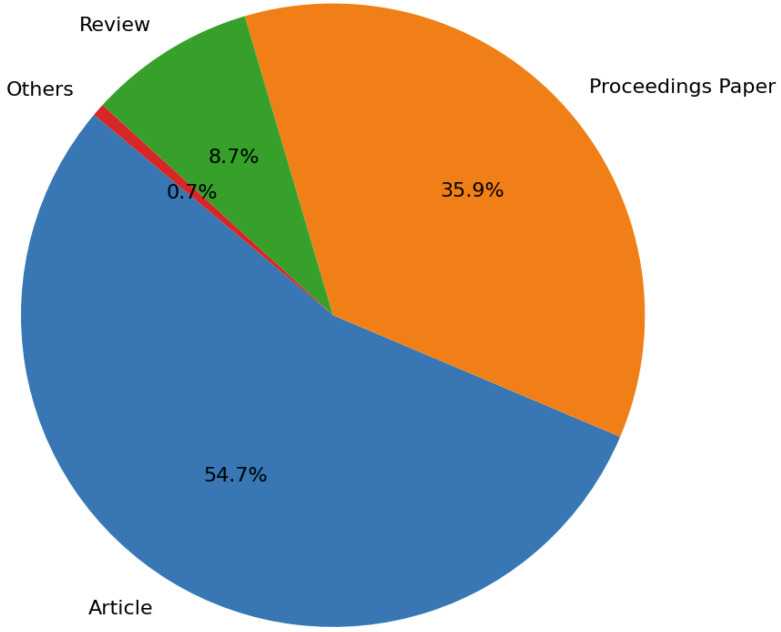
Distribution of document types.

The analysis of publication trends in Big Data and IoT presented in Figure 3 reveals a landscape dominated by original research with 54.7% of contributions categorized as Articles. This indicates that researchers are actively generating new knowledge and driving innovation in these evolving fields. Additionally, 35.9% of the publications are Proceedings Papers, which highlight the essential role of conferences in facilitating knowledge exchange and collaboration among professionals.

The representation of Review articles, at 8.7%, indicates a potential opportunity for researchers to undertake systematic reviews or meta-analyses that synthesize existing knowledge and provide comprehensive overviews to guide future inquiries in Big Data and IoT research. Furthermore, the minimal 0.7% in the “Others” category emphasizes a clear focus within the research community on traditional publication formats.

Overall, these findings illustrate a dynamic research environment, showcasing the importance of original contributions while also highlighting the need for more synthesized knowledge to support ongoing and future inquiries in Big Data and IoT.

**Figure 4:**
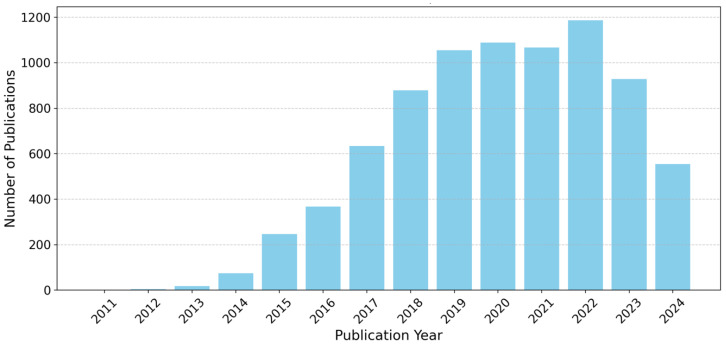
Number of publications per year.

In Figure 4, the number of publications from each year can be observed. It illustrates patterns in research output over the years, with a notable peak in 2022, indicating a surge in interest and innovation in Big Data and IoT. This increase may be linked to technological advancements or emerging trends. The subsequent decline from 2022 to 2024 raises questions about influencing factors, while the steady growth observed from 2013 to 2020 reflects increasing investment and an expanding research community.

**Figure 5:**
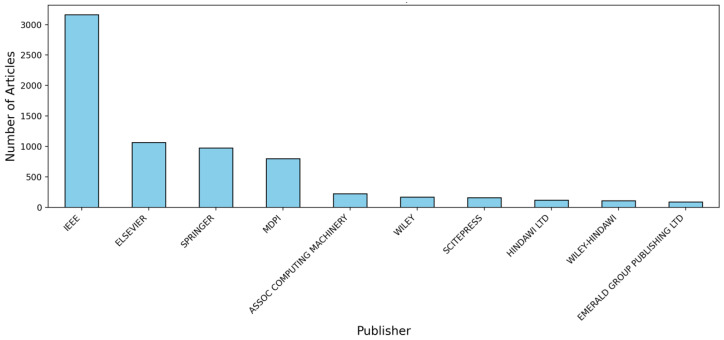
Number of articles per publisher.

Figure 5 highlights a disparity in the number of articles published across different journals, showing that over 3000 articles have been published in IEEE journals. In comparison, Elsevier, Springer, and MDPI each account for between 500 and 1000 articles, while other publishers contribute fewer than 500 articles. This distribution is significant for the article as it underscores the dominant role of IEEE in the fields of technology and engineering research.

**Figure 6:**
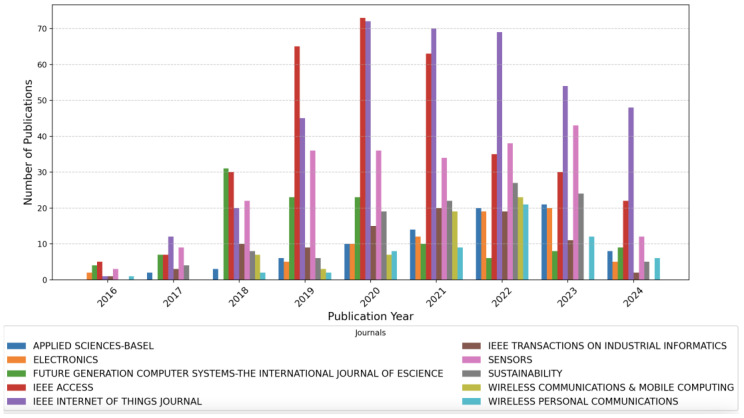
Top 10 publication trends by journals.

Figure 6 illustrates the evolution of publications in the top journals publishing on IoT and Big Data topics from 2016 to 2024. The data reveal minimal contributions across journals prior to 2016, which is followed by a significant rise in publications. Between 2018 and 2020, publication numbers peaked with journals like *IEEE Internet of Things Journal* and *Sensors playing* prominent roles. Similarly, *IEEE Access* and *Wireless Communications* experienced substantial growth. Post-2020, the trends stabilize, with journals such as *Applied Sciences-Basel* and *Sustainability* maintaining significant contributions. This trend indicates a growing interest in fields related to wireless communications, sustainability, and IoT over recent years.

**Figure 7:**
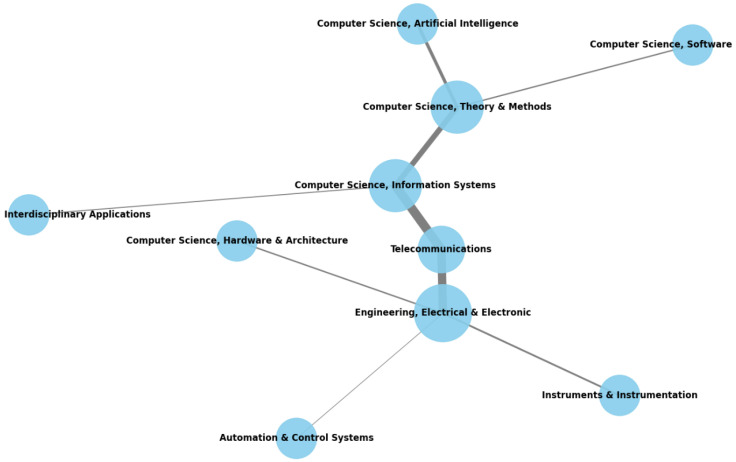
WoS categories network connections.

Figure 7 showcases WOS categories network connections. Each node represents a specific research category, and the size of the nodes reflects the degree of co-occurrence of the respective category with others. The connections or edges between the nodes illustrate how often pairs of categories appear together within the same literature.

For instance, categories like “Telecommunications” and “Engineering, Electrical & Electronic” are prominently connected, indicating an overlap in research between these areas. Other closely linked fields, such as “Computer Science, Information Systems” and “Computer Science, Theory & Methods”, show similar co-occurrence patterns. The visualization highlights key interdisciplinary research clusters, particularly around electrical engineering and computer science topics, where the fields most commonly intersect.

This interconnectedness underscores the significance of interdisciplinary collaborations in driving IoT and Big Data research forward. Our findings emphasize that such collaborations are significant for addressing complex challenges and fostering innovation. Fields such as computer science and engineering, which dominate the co-occurrence network, converge to develop technologies for real-time data processing and scalable IoT frameworks, which are capabilities essential for applications like industrial IoT and smart cities. These collaborations enable advancements in system efficiency, data integration and scalability, addressing the practical demands of these domains. By bridging expertise across categories, as illustrated in Figure 7, the research highlights how interdisciplinary research strengthens the foundation for transformative solutions in IoT and Big Data.

**Figure 8:**
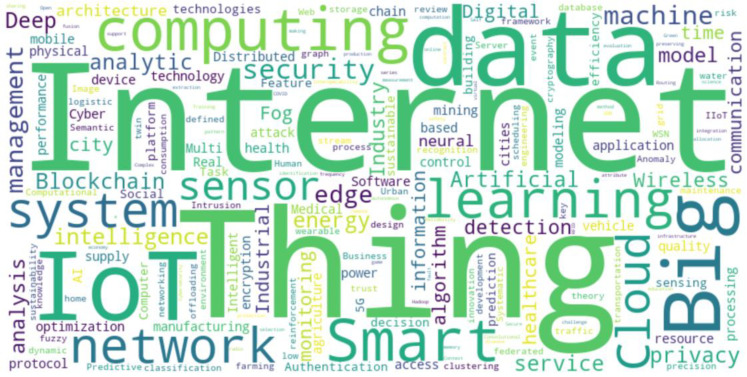
Word cloud graphic representation of the main keywords.

The word cloud presented in Figure 8 highlights the most frequently occurring terms in the analyzed publications with “Internet”, “IoT Thing”, “Big Data”, “network” and “computing” standing out as the dominant keywords. These terms reflect the core areas of focus within the research landscape of Big Data and IoT, indicating the primary technological components and concepts that drive innovation in these fields. For instance, “Internet” and “IoT Thing” emphasize the importance of connectivity and the integration of physical devices in the Internet of Things, while “Big Data” and “computing” underscore the role of large-scale data processing and computational power in supporting these systems.

**Figure 9:**
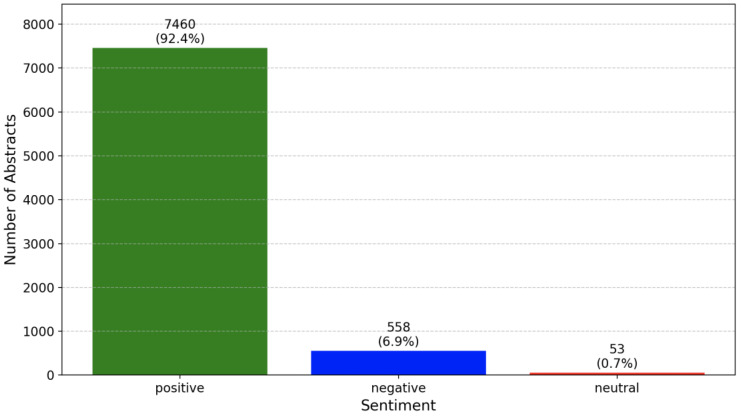
Sentiment analysis of abstracts.

The sentiment analysis of abstracts, as shown in the bar chart in Figure 9, reveals a clear dominance of positive sentiment within the dataset. Over 7000 abstracts are classified as positive, while fewer than 1000 are identified as negative. Neutral abstracts constitute a negligible fraction in comparison. This distribution aligns with the typical purpose of abstracts, which is to emphasize the significance, advancements, and optimistic outcomes of the research, reflecting a generally positive tone in Big Data and IoT studies. Positive abstracts focus on showcasing innovations, solutions to existing challenges or promising results that highlight the contributions of the research.

Negative sentiments, on the other hand, primarily arise from discussions of unresolved issues or barriers in the field. These include challenges such as the limited scalability of existing frameworks, the lack of robust interoperability solutions, and ongoing difficulties in efficiently processing large volumes of real-time data. Such abstracts often emphasize the limitations or risks that impede the broader adoption or application of IoT technologies.

Neutral sentiments are linked to more descriptive or method-focused studies that neither highlight advancements nor identify major challenges. These abstracts frequently appear in conference publications, which may lack in-depth analysis or the practical validation of proposed solutions. Additionally, gaps in systematic review studies lead to fragmented knowledge, making it difficult to synthesize a comprehensive understanding of progress in the field.

**Figure 10:**
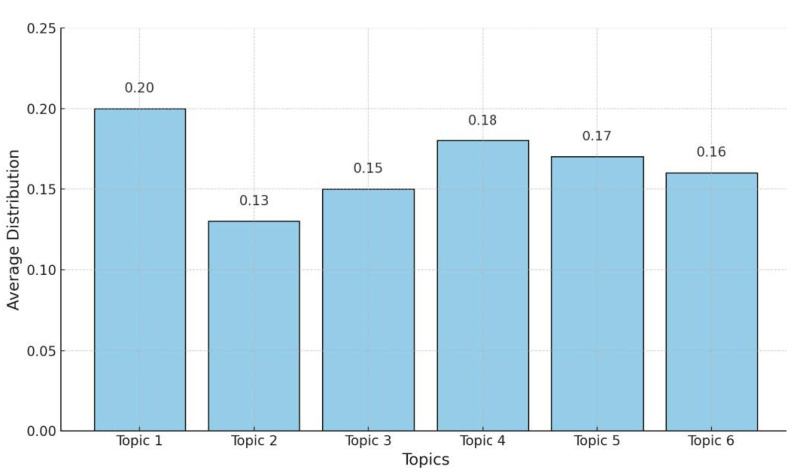
Average topic distribution.

Figure 10 displays the average distribution of six topics extracted from the Abstract column. Topic 1 has the highest average distribution, approximately 20%, suggesting that themes related to “smart systems”, “IoT”, and “data” are the most frequently discussed in the dataset. Topic 4 follows closely, with an average distribution of around 18%, highlighting a focus on areas like “IoT”, “cloud computing”, and “edge computing”. Topics 5 and 6, with average distributions of about 17% and 16% respectively, indicate attention toward “security models”, “learning systems”, and “industrial IoT applications.” On the other hand, Topic 2 has the lowest average distribution, around 13%, suggesting that themes related to “data processing”, “sensors”, and “proposed technologies” are less prominent in this dataset. Topic 3, with an average distribution of 15%, reflects research areas such as “Big Data”, “healthcare applications”, and “IoT in healthcare”. Therefore, Figure 10 illustrates how the identified topics are proportionally distributed across the dataset. The average distributions indicate the extent to which each topic is covered in the analyzed documents. A higher average distribution, as seen in Topic 1, suggests that the themes associated with this topic are more frequently discussed and appear in more documents. Conversely, lower values, such as those for Topic 2, indicate that these topics are less frequently present in the dataset.

The six identified topics overlap in several areas (as in Table 3), reflecting shared themes and dependencies across research areas. For instance, Topic 1 focuses on “smart systems” and “IoT”, which require the infrastructure addressed in Topic 4, such as “cloud computing” and “edge computing”, for data management and processing. Topic 3, centered on “healthcare applications”, is closely related to Topic 6, which addresses “learning systems” that are essential for analyzing healthcare data and enabling predictive solutions. Topics 4 and 5 are also linked, as “security models” from Topic 5 are critical for securing cloud and edge computing frameworks described in Topic 4. Although Topic 2 has the smallest distribution, its focus on “data processing” and “sensors” serves as a foundational element that supports the systems described in the other topics.

**Figure 11:**
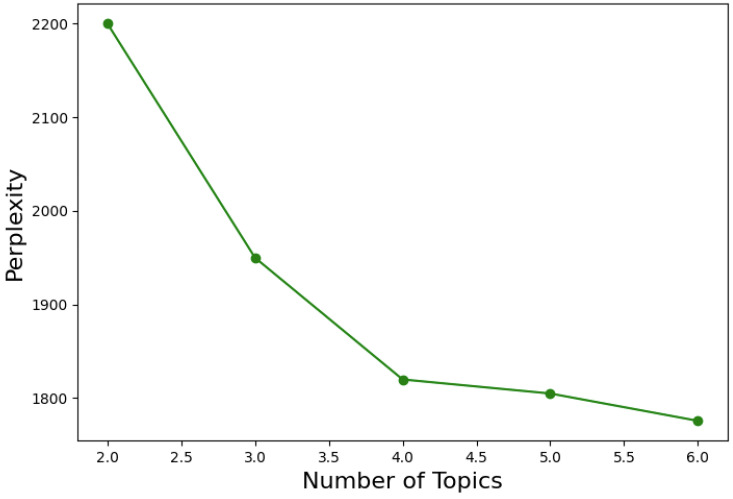
Perplexity vs. number of topics.

The perplexity plot, presented in Figure 11, helps evaluate the quality of the LDA model by plotting the perplexity values across different numbers of topics. As shown in the graph, the perplexity decreases consistently as the number of topics increases from two to six with the lowest perplexity achieved at six topics. This indicates that six topics provide the best fit for this dataset. The steady decline in perplexity suggests that increasing the number of topics improves the model’s ability to capture the structure of the dataset without signs of overfitting.

**Figure 12:**
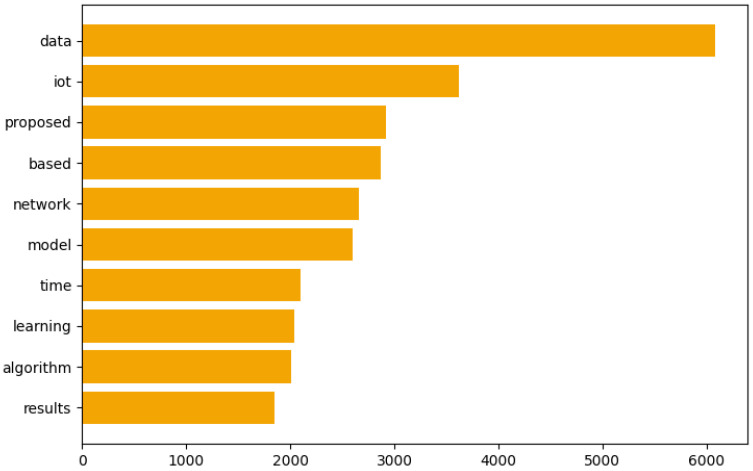
Topic 1 word distribution.

In Figure 12, the bar chart illustrates the most important words within Topic 1. Words like “data”, “IoT”, “proposed” and “network” are prominent, reflecting the central themes of IoT and Big Data in this topic.

**Figure 13:**
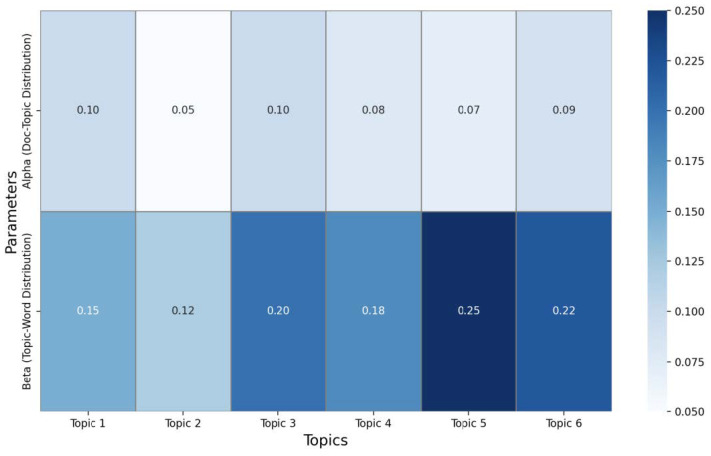
Alpha and beta parameters.

The heatmap presented in Figure 13 visualizes the alpha and beta parameters across six topics, providing insights into their distribution and significance. The alpha parameter controls the document–topic distribution, indicating how frequently a topic appears across the dataset. Higher alpha values suggest that a larger number of documents are likely to include these topics, highlighting their relevance within the dataset. Topics 1, 3 and 6 exhibit relatively higher alpha values, suggesting their significance and broader coverage across the collection of dataset analyzed. The beta parameter, on the other hand, influences the topic–word distribution, reflecting the diversity of words associated with each topic. A higher beta value signifies that a topic encompasses a broader and more diverse vocabulary, making it less narrowly focused. Among all topics, Topic 5 has the highest beta value, indicating that it includes the most varied range of words. This makes Topic 5 more diverse in scope compared to the other topics, potentially representing a broader or more general theme within the dataset.

The visual contrast in the heatmap, represented by the intensity of the color gradient, emphasizes these differences. For instance, the darker blue shading for Topic 5 in the beta row highlights its significantly higher value, underlining its diversity. In contrast, the lighter shades for alpha in Topics 2 and 4 demonstrate their relatively lower values, indicating that these topics are less prevalent across the dataset.

Table 4 reflects the distribution of six main topics extracted from an LDA model applied to the analyzed dataset. Each topic is associated with three key values: the average distribution of the topic across the entire dataset, the alpha parameter, and the beta parameter. The average distribution shows how frequently each topic appears in the analyzed texts. In this case, Topic 3 and Topic 1 have the highest distributions, both around 0.246, indicating that these topics are predominant in the dataset. In contrast, Topic 2 has the lowest distribution at 0.1438, suggesting that this topic is less frequently discussed in the abstracts.

The alpha parameter controls how spread out the topics are within each document. Higher alpha values, such as those for Topic 1 and Topic 3 (both with a value of 0.10), indicate that dataset contains a more balanced proportion of multiple topics. On the other hand, Topic 2, with an alpha value of 0.05, appears in fewer datasets and tends to be present in smaller proportions within the texts where it is included.

The beta parameter refers to the distribution of words within each topic. A higher beta suggests that the respective topic is composed of a wider range of words. For example, Topic 5, with a beta of 0.25, includes a broader variety of terms compared to Topic 1, which has a lower beta of 0.15, indicating a more limited and focused vocabulary centered around specific terms.

**Figure 14:**
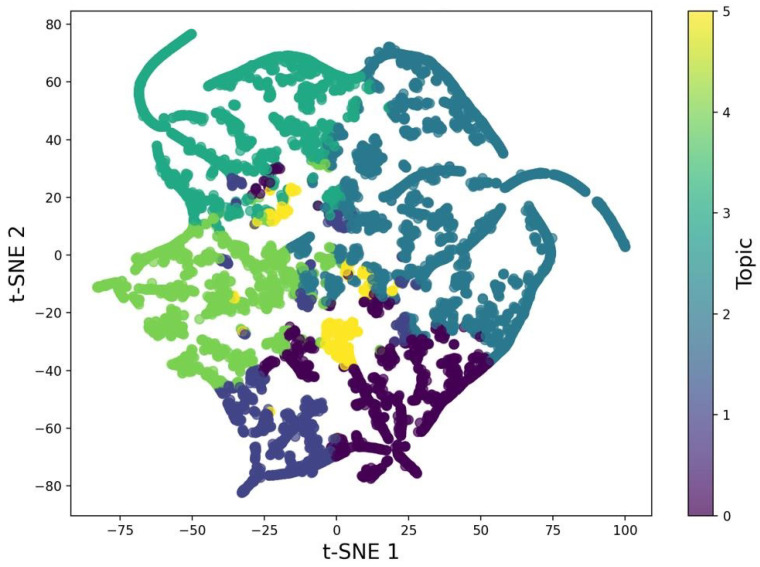
A 2D t-SNE projection of topics.

In Figure 14, a visual representation of topics derived from the abstracts of research papers is presented. Using LDA, the content of these abstracts was grouped into six distinct topics, and t-SNE (t-distributed Stochastic Neighbor Embedding) was applied to project these high-dimensional topic distributions into a two-dimensional space. This makes it easier to visually understand the relationships and distinctions between the topics.

In the graph, each region corresponds to a different topic with transitions between colors showing how some documents may overlap across multiple themes. For example, articles that discuss both the Internet of Things (IoT) and Big Data might lie in areas where the colors blend, while articles focusing solely on one of these subjects are placed in more distinct regions.

**Figure 15:**
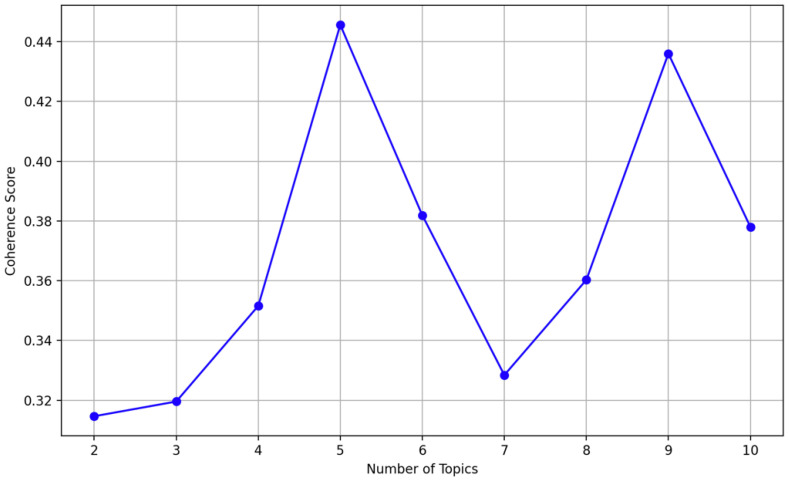
Coherence according to the number of topics.

From Figure 15, a steady rise in coherence from two topics (0.32) to a peak at five topics (0.44) is noticed, suggesting that the topics at this point are the most interpretable and meaningful. After the peak, the coherence score drops at six topics (0.36), followed by fluctuations, reaching another high point at nine topics (0.41).

**Figure 16:**
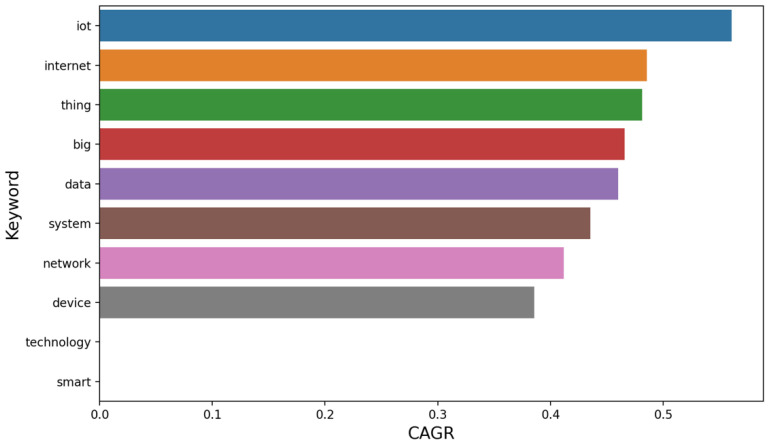
Abstract keywords evolution over time.

Figure 16 illustrates the compound annual growth rate (CAGR) for the top keywords, showing how their usage has evolved over time. CAGR represents the rate at which a value grows annually over a specific period. In this case, the keyword “IoT” shows the highest growth, indicating that it has become increasingly common in the analyzed dataset. The terms “internet” and “thing” follow, also reflecting significant increases in usage. Keywords like “big”, “data”, “system” and “network” have grown at a steady pace, while “device”, “technology” and “smart” exhibit lower growth rates compared to the others.

**Figure 17:**
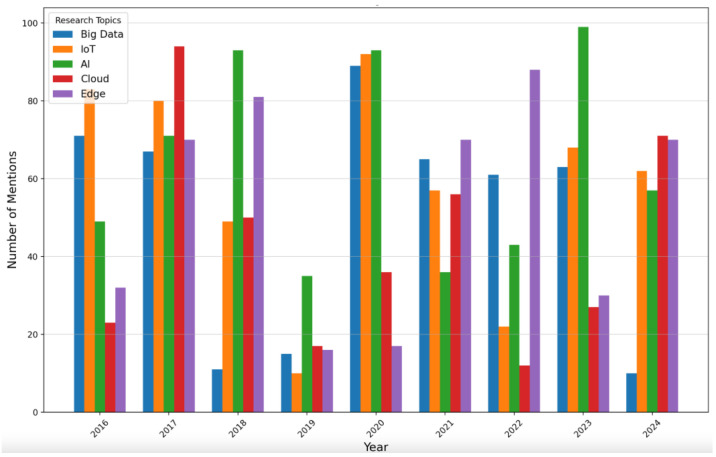
Number of mentions over time.

Figure 17 illustrates the trends of Big Data, IoT, AI, Cloud and Edge. The x-axis denotes the years, while the y-axis represents the count of mentions, providing an intuitive visualization of how each topic’s popularity has fluctuated over time. Each year is represented as a group of bars with each bar in the group corresponding to one of the five research topics. Certain topics such as “AI” and “IoT” show consistent activity with peaks in specific years that suggest heightened research focus or breakthroughs. Topics like “Edge” and “Cloud” demonstrate periodic surges, indicating their growing importance in recent years. On the other hand, “Big Data” maintains a relatively stable trend, highlighting its sustained relevance throughout the period.

Table 5 presents the top terms and their respective weights across six different topics. Each row represents a specific topic, and within each row, the “Score” columns combine the key terms with their associated weights, providing an understanding of the importance of each term within the topic. For instance, Topic 0 is characterized by terms like “data” (0.011), “IoT” (0.009), and “technologies” (0.009), suggesting a focus on technologies and IoT. Similarly, Topic 1 highlights “smart” (0.012) and “data” (0.009) as central concepts, indicating discussions around smart technologies and their data integration. Across all topics, key themes related to data, IoT, and technologies are notable, reflecting their significance in the broader discourse. The weights attached to each term reflect the strength of their association within each topic, allowing for a more detailed understanding of the underlying thematic structure (as in Table 5).

In the following, the six topics generated from the dataset will be analyzed in detail, using the pyLDAvis tool for topic modeling visualization. pyLDAvis is an interactive tool that provides a clear, visual representation of topics created using LDA. The tool helps explore relationships between the topics and highlights the most relevant terms for each. This complements the earlier table, “Key Terms and Their Weights Across Topics,” by offering an interactive and detailed view of the term distribution and the thematic structure within the dataset.

LDA, a widely used topic modelling algorithm, was applied to extract latent topics from the dataset. This probabilistic approach identifies groups of co-occurring terms that frequently appear together, revealing distinct themes embedded in the data. The number of topics (6) was determined through iterative experimentation. Topic coherence, a metric used to measure the semantic consistency of topics, was a key determinant in selecting the optimal number of topics. A higher coherence score (0.79) suggests that the terms within each topic are meaningfully related, enhancing the quality of the topics.

**Figure 18:**
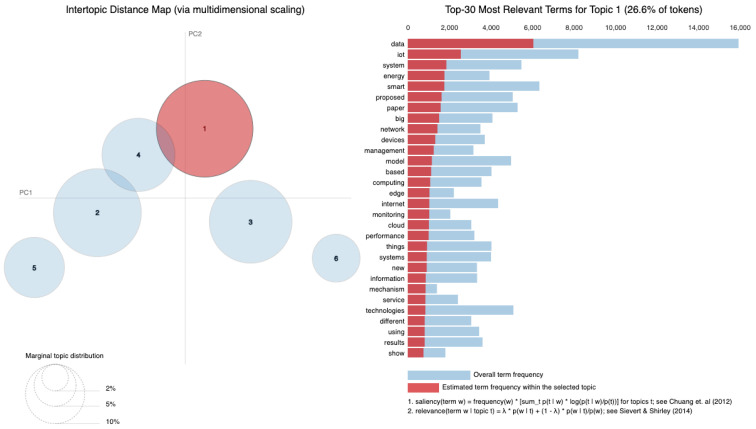
PyLDAvis: the first topic.

Figure 18 focuses on Topic 1, the most dominant one, as shown by the larger red circle on the left side of the visualization. The intertopic distance map (on the left) shows the proximity of different topics with larger circles representing the most common topics in the dataset. Topic 1 stands out as a significant theme, indicated by its larger circle and central position, suggesting it covers a broad range of discussions in the dataset.

On the right side, the top 30 most relevant terms for Topic 1 are displayed. Words like “data”, “IoT”, “system” and “energy” are among the most frequent, signaling that Topic 1 revolves around data systems and IoT technologies, likely in the context of smart systems and energy-related applications. The bar chart highlights the relevance of these terms within the topic (red portion) and compares their overall frequency in the entire corpus (blue portion).

**Figure 19:**
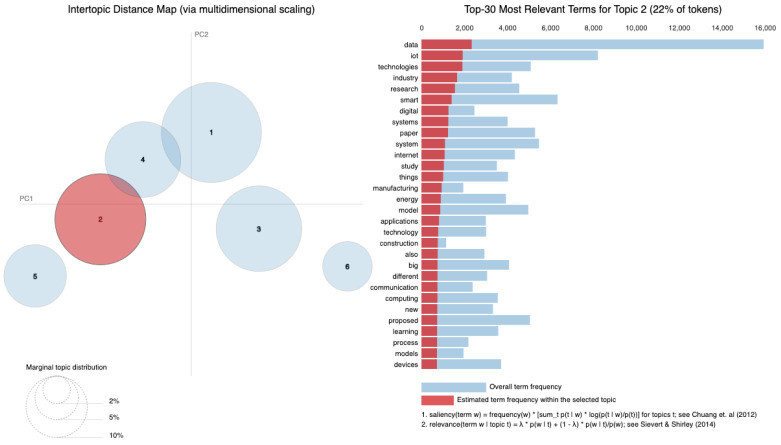
PyLDAvis: the second topic.

Topic 2, presented in Figure 19, focuses on the application of data and IoT technologies, as indicated by terms such as “technologies”, “industry” and “research”. This topic deals with how IoT and related technologies are transforming various industries, including manufacturing and energy sectors. Terms like “manufacturing” and “energy” suggest that industrial processes and energy management are key areas of focus within this topic. The presence of terms like “systems”, “model” and “applications” hints at the technical and applied nature of the research, pointing toward discussions around the development and deployment of models and systems in practical settings.

**Figure 20:**
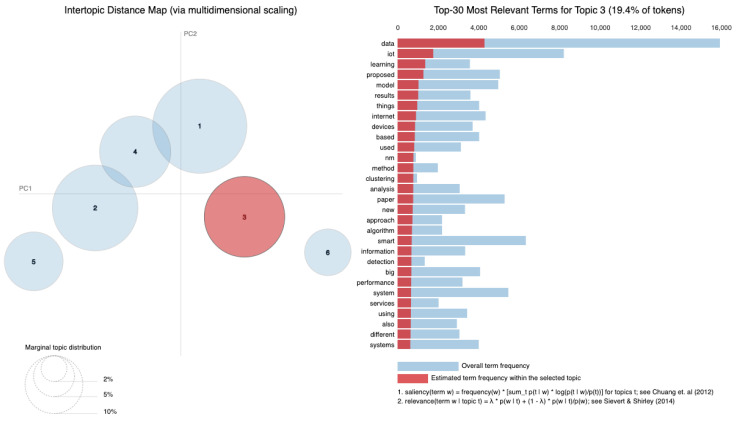
PyLDAvis: the third topic.

The third topic (in Figure 20) emphasizes terms like data, IoT, and learning, indicating a focus on machine learning and IoT applications. The prominence of terms such as “proposed”, “model” and “results” suggests that this topic is oriented toward research involving new methods and models in the IoT domain, which is related to learning algorithms or clustering techniques. The appearance of terms like “clustering”, “algorithm” and “detection” highlights the technical focus on processing and analyzing data within IoT systems.

**Figure 21:**
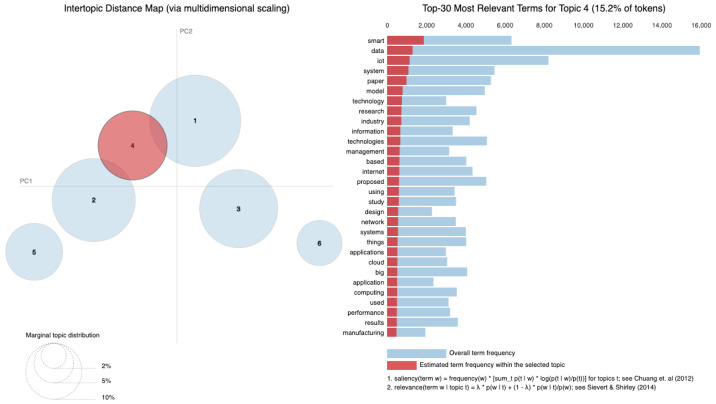
PyLDAvis: the fourth topic.

The fourth topic (in Figure 21) highlights terms such as smart, data, and IoT, with “smart” being the main term, suggesting a focus on smart technologies and systems. The presence of terms like “system”, “model” and “network” points to discussions surrounding smart systems, their applications, and management. Additionally, terms like “technology” and “research” reflect an ongoing focus on technological advancements and their implications in various industries, including manufacturing and network systems.

**Figure 22:**
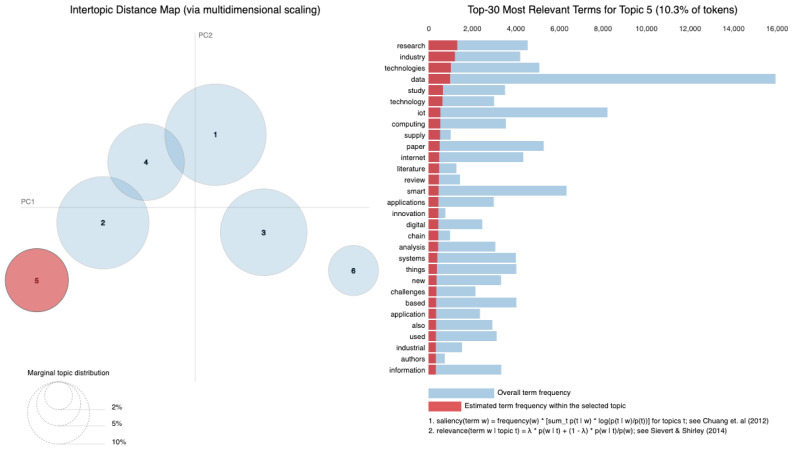
PyLDAvis: the fifth topic.

Topic 5, presented in Figure 22, highlights the intersection of research and industry, focusing on technologies, data, and innovation. It also touches upon the role of digital chains and supply systems, suggesting an industrial focus on digital transformation. The presence of terms like “applications”, “analysis” and “innovation” underscores the practical implications of these technologies within industry settings.

**Figure 23:**
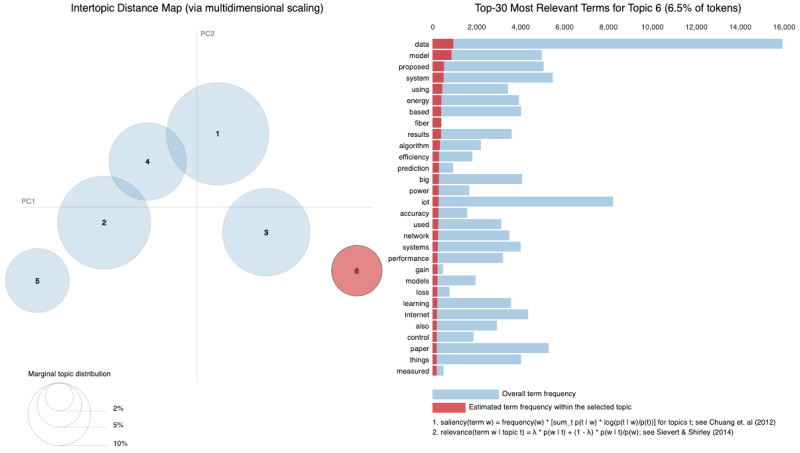
PyLDAvis: the sixth topic.

Figure 23 presents the last topic, Topic 6, which focuses on technical aspects such as data modeling, system performance, and prediction algorithms. It delves into the efficiency of IoT networks with terms like “accuracy”, “power” and “performance” standing out, indicating the attention to optimization in IoT infrastructures. Additionally, this topic emphasizes the use of algorithms to enhance system control and accuracy, showcasing the depth in IoT performance improvements.

Figure 18, Figure 19, Figure 20, Figure 21, Figure 22 and Figure 23 also illustrate how these topics blend in research that spans multiple domains with transitions between themes indicating overlap in areas such as network efficiency, real-time analytics, and scalable architectures. This interconnectedness underscores how advancements in one topic, such as machine learning techniques from Topic 3, can enhance the predictive capabilities discussed in Topic 6 or the operational efficiency highlighted in Topics 2 and 5.

## 5. Conclusions

Bearing in mind the goals formulated in the introduction, we extracted the following main findings:

- Trends over the years indicate a marked increase in publication output, peaking in 2022. This surge is likely linked to rapid technological advancements and heightened interest in these fields. Nevertheless, the decline observed from 2022 to 2024 prompts further investigation into the factors influencing research momentum, suggesting a potential plateau in interest or resource allocation.
- The analysis of publications by source reveals a striking concentration within IEEE journals, which dominate the field with over 3000 articles published. This dominance underscores IEEE’s significant role in advancing technology and engineering research. In contrast, other publishers, including Elsevier, Springer and MDPI, contribute substantially fewer articles, indicating a potentially skewed landscape that favors specific publication venues.
- The visualization of keyword co-occurrences and sentiment analysis demonstrates the interdisciplinary nature of research in Big Data and IoT with strong links between telecommunications, engineering and computer science. The prevalent positive sentiment across the abstracts suggests that the prevailing research narrative is optimistic, highlighting advancements and beneficial trends within these fields.
- The topic modeling using LDA indicates six prominent themes, including smart systems, industrial applications and machine learning, illustrating the multifaceted focus of current research. The evolving usage of key terms over time, particularly the rise of “IoT”, reflects the growing integration of IoT concepts into various technological domains. The PyLDAvis visualizations analyze six main topics within a dataset each with unique thematic focuses. The first topic is the most dominant, centered on data systems and IoT technologies, particularly in smart systems and energy applications, with frequent terms like “data”, “IoT”, “system” and “energy.” The second topic explores IoT applications in various industries, specially manufacturing and energy, highlighting terms like “technologies”, “industry” and “research”. The third topic emphasizes IoT and machine learning, focusing on new methods and models involving algorithms and clustering techniques. The fourth topic centers on smart technologies, discussing systems, models, and networks, with terms such as “smart” and “system”. The fifth topic examines the intersection of research, technology, and industry, focusing on digital transformation in supply systems and innovation. The sixth topic delves into technical IoT aspects like system performance and prediction algorithms with an emphasis on optimizing IoT infrastructure for accuracy and control. Each topic’s terms and themes illustrate different facets of IoT and data technologies, ranging from technical implementations to industry applications.

LDA has several limitations that can influence the reliability and interpretability of our analysis on Big Data and IoT research. One of the main challenges is its sensitivity to hyperparameters, such as the number of topics (K). The choice of K significantly affects the coherence and distinctiveness of topics. In this research, the selection of six topics was optimized tuning the hyperparameters of the LDA model in order to identify the best K. Another issue with LDA is its reliance on probabilistic word distributions rather than deep semantic understanding. The model does not differentiate between synonymous terms or recognize broader conceptual relationships. Consequently, similar concepts, such as “predictive maintenance” and “fault detection”, might be assigned to separate topics despite their close thematic connection. Despite these limitations, LDA is a valuable tool for extracting overarching themes in research. However, to enhance the robustness of the analysis, our future research will explore alternative approaches, such as BERTopic or neural topic models like BERT-LDA to offer better semantic understanding.

Our current research limited its dataset to Web of Science publications due to the platform’s well-established reputation for indexing high-quality, peer-reviewed literature and its comprehensive coverage across a wide range of disciplines.

In conclusion, while the landscape of Big Data and IoT research is marked by dynamic innovation and significant original contributions, it also presents notable gaps in synthesized knowledge that warrant attention. Future researchers should focus on developing comprehensive review articles that could bridge these gaps, guiding subsequent inquiries. The ongoing evolution of publication types and trends emphasizes the need for adaptability in research dissemination strategies, ensuring that diverse formats are utilized to effectively communicate findings to a broader audience.

## Figures and Tables

**Table 1:** Comparison of previous studies.

Ref	Objectives	Methods	Keywords Focus	Main Findings
[6]	Overview of advancements in IoT, including complex networks, SIoT, and Big Data analytics.	Bibliometric analysis focusing on new IoT technologies and integration of SIoT and Big Data.	IoT, Big Data, social IoT, complex networks, research trends.	Highlights emerging technologies like SIoT and Big Data analytics, and the future of IoT in optimizing data processing and enhancing automation.
[7]	Highlight recurring themes in IoT and Big Data research.	Literature review of MDPI and IEEE Xplore analyses.	IoT, Big Data, recurring themes.	Recurring themes include real-time data processing and smart applications.
[8]	Scientific mapping of IoT research landscape.	Bibliometric analysis using SciMAT.	IoT, research landscape, SciMAT.	Identified growth areas and potential in IoT research.
[9]	Analyze machine learning research trends using bibliometrics.	Bibliometric methods using VOSviewer and Bibliometrix.	Machine learning, trends, collaboration.	Visualized trends and influential authors in machine learning.
[10]	Review trends in intrusion detection systems for IoT.	Bibliometric analysis and network analysis.	Intrusion detection, IoT, network analysis.	Highlighted trends in IoT security and machine learning for IDS.
[11]	Identify themes, collaborations in Big Data research.	Co-word analysis and VOSviewer mapping.	Big Data, themes, collaborations.	Big Data intersect with multiple domains like healthcare and finance.
[12]	Assess IoT applications in smart agriculture.	Co-citation and keyword co-occurrence analysis.	Smart agriculture, IoT, sustainability.	IoT applications in agriculture have grown significantly since 2017.
[13,14]	Discuss ethical implications of IoT and Big Data.	Ethical discussion and thematic review.	Ethical implications, privacy, security.	IoT integration raises ethical and data security concerns.
[15]	Identify research gaps in IoT interoperability and ML.	Bibliometric analysis of research gaps.	Interoperability, machine learning.	Research gaps in interoperability and advanced ML frameworks.
[16]	Study IoT and Big Data in smart city development.	Cluster analysis of publication trends.	Smart cities, energy, traffic optimization.	Big Data and IoT improve urban planning and resource allocation.
[17]	Proposed an Edge–Fog–Cloud architecture to process IoT and smart metering data.	Hybrid architecture combining edge, fog, and cloud computing layers.	IoT, Edge computing, Fog computing, Cloud computing, smart metering.	Enhances system efficiency and reduces latency by distributing processing tasks across Edge, Fog, and Cloud layers.
[18]	Examines the integration of IoT in logistics and supply chain management, focusing on innovation and optimization.	Bibliometric analysis of published IoT research in logistics.	IoT, logistics, supply chain management, Big Data optimization.	Highlights key areas of innovation in IoT for logistics, particularly big data optimization and food supply chain applications.
[19]	Investigates trends in education Big Data and learning analytics.	Bibliometric analysis of education-related Big Data and analytics.	Education, Big Data, learning analytics.	Provides trends and recommendations for improving educational outcomes using Big Data and analytics.
[20]	Identifies research directions and trends in the fast-evolving Big Data field.	Bibliometric mining of Big Data research.	Big Data, research trends, bibliometric analysis.	Describes emerging research trends and opportunities within the Big Data field.
[21]	Analyzes Big Data research related to value creation and capture.	Bibliometric analysis focusing on Big Data’s potential for business value.	Big Data, value creation, value capture.	Assesses the impact of Big Data on value creation and business strategies.
[22]	Visualizes research trends in the field of home IoT.	Bibliometric analysis using VOSviewer for trend visualization.	Home IoT, smart homes, IoT research trends.	Provides insights into key trends and future directions in home IoT research.
[23]	Explores research on edge computing applications in IoT.	Bibliometric analysis of edge computing research.	Edge computing, IoT, security, privacy.	Highlights the critical role of edge computing in IoT systems, focusing on security, trust, and privacy.
[24]	Map IoT and Big Data in smart infrastructure.	Mapping publication trends using Web of Science.	Smart infrastructure, IoT, grids.	IoT enhances smart grids and urban resilience.
[25]	Survey Big Data challenges in IoT ecosystems.	Survey of Big Data management frameworks.	Big Data, IoT, architectures.	Big Data frameworks address IoT scalability and security.
[26]	Highlight cross-disciplinary challenges in IoT and Big Data.	Thematic analysis of interdisciplinary challenges.	Cross-disciplinary IoT challenges.	IoT research requires cross-disciplinary solutions.
[27]	Study IoT in healthcare, wearable technology focus.	Bibliometric review of wearable IoT.	Healthcare IoT, wearable technology.	Wearable IoT devices support chronic disease management.
[28]	Analyze IoT in precision agriculture for sustainability.	Keyword analysis and bibliometric methods.	Precision agriculture, climate, IoT.	IoT optimizes agricultural practices and climate adaptation.
[29]	Explore predictive maintenance using industrial IoT.	Predictive maintenance bibliometric review.	Industrial IoT, predictive maintenance.	IoT improves industrial automation and asset management.
[30]	Compare regional trends in IoT applications in education.	Bibliometric comparison of IoT in education.	IoT in education, regional trends.	IoT in education emphasizes regional differences in application.
[31]	Highlight evolving trends in IoT and Big Data research.	Systematic review of bibliometric studies.	IoT, Big Data, thematic clusters.	IoT and Big Data have expanded across diverse domains.
[32]	Analyze IoT in agriculture, emphasizing food security.	Keyword and thematic analysis of agriculture IoT.	IoT, agriculture, food security.	IoT aids in food security and precision agriculture.
[33]	Explore Industry 4.0 technologies in agriculture.	Keyword analysis and Industry 4.0 mapping.	Industry 4.0, agriculture, IoT.	Industry 4.0 enhances agricultural data management.
[34,35]	Study IoT in industrial automation and efficiency.	Cluster analysis and bibliometric review.	Industrial IoT, automation, maintenance.	IoT predictive maintenance reduces industrial downtime.
[36]	Examine IoT’s impact on customer engagement models.	Analysis of IoT-driven customer models.	Customer engagement, IoT, personalization.	IoT drives customer personalization and secure transactions.
[37]	Highlight IoT’s role in environmental sustainability.	Bibliometric analysis of sustainability applications.	Sustainability, IoT, resource conservation.	IoT contributes to environmental monitoring and energy conservation.
[38]	Focuses on anomaly detection in electricity consumption using machine learning and Big Data.	Machine learning algorithms combined with Big Data analysis.	Anomaly detection, machine learning, Big Data, electricity consumption.	Identifies anomalies in electricity consumption, improving fraud detection and system efficiency using Big Data analytics and machine learning.
[39]	Analyze IoT in healthcare focusing on real-time monitoring.	Bibliometric study of healthcare IoT.	Healthcare IoT, wearable technologies.	IoT enhances patient care but faces privacy and interoperability issues.
[40,41]	Identify IoT and Big Data trends in sustainable agriculture.	Precision agriculture keyword analysis.	Sustainability, agriculture, IoT.	IoT drives sustainable agriculture and resource efficiency.
[42]	Explore applications of WSNs and IoT in Industry 4.0.	Systematic lliterature review	Industry 4.0, IoT, WSN.	IoT and WSN improve industrial efficiency, enabling real-time monitoring and data-driven decision making.
[43]	Analyze global trends in smart homes for older adults.	Bibliometric and scientometric analysis	Smart homes, older adults, IoT, aging.	Technologies address health monitoring and assistive living needs for aging populations.
[44]	Investigate smart technologies for sustainable urban water management.	Urban analysis	Smart technologies, water management, IoT.	IoT supports efficient water resource management, promoting sustainability.
[45]	Examine IoT innovations for indoor air quality monitoring.	Systematic review, bibliometric analysis	IoT, air quality, indoor environment.	IoT enhances real-time environmental health monitoring, benefiting health outcomes.
[46]	Map a decade of research on smart homes for elderly using scientometric methods.	Scientometric review, CiteSpace	Smart homes, elderly, research trends.	Insights highlight focus on fall detection, health, and elderly comfort in smart home designs.
[47]	Develop smart building strategies to enhance safety and health for the elderly.	Systematic and bibliometric analysis	Smart buildings, elderly, safety, health.	Smart building designs address safety risks and promote elderly health and well-being
[48]	Analyze IoT privacy and security challenges in smart homes.	Analytical review	IoT, privacy, security, smart homes.	Privacy issues in IoT require robust security measures to ensure user trust in smart home systems.
[49]	Review smart home and city developments concerning sustainability and future trends.	Comprehensive review	Smart homes, smart cities, sustainability.	Integration of sustainable concepts in urban and residential IoT systems is key for future growth.
[50]	Systematically analyze trends and recommendations for smart homes.	Systematic analysis	Smart homes, trends, IoT.	Highlights future directions in smart home innovations, emphasizing user-centric and secure designs.
[51]	To explore the conceptualization of smart cities in the context of education, identifying how education-related themes are integrated into smart city frameworks.	Co-word analysis, thematic clustering, keyword co-occurrence analysis	Smart cities, education, urban development, conceptualization.	Education as a central component of smart city research, offering thematic maps of the field’s evolution.
[52]	To explore the intellectual development of smart city research using bibliometric and main path analysis.	Bibliometric analysis, citation and co-citation analysis, main path analysis	Smart cities, research evolution, citation networks, main path analysis.	Evolution of smart city research, identifying key works and research pathways. It emphasizes the intellectual flow of ideas within the smart city domain.
[53]	To analyze the role of artificial intelligence (AI) in smart cities, focusing on trends and relationships between key works in this area.	Citation analysis, bibliographic coupling, keyword co-occurrence	AI, smart cities, urban management, machine learning.	Integration of AI into smart cities, providing insights into the technologies and applications that are transforming urban management.
[54]	To provide a bibliometric analysis of smart cities research, identifying key trends, research clusters, and major contributors.	VOSviewer, CiteSpace, co-authorship analysis, keyword co-occurrence	Smart cities, co-authorship, research trends, interdisciplinary collaboration.	Rapid growth in publications with emerging themes like AI in governance.
[55]	To provide a bibliometric analysis of scientific publications and patents on smart cities, bridging academic research with industrial applications.	Bibliometric analysis, patent analysis, vantagepoint	Smart cities, patents, scientific publications, industry applications.	Importance of the link between academic research and industrial innovations.

**Table 2:** Methodology flow using parameters.

	Step	Description	Details
1	Setting Objective	Analyze the main themes and sentiment across a dataset of IoT and Big Data publications.	Time Frame: Various years of publication. Data Source: Web of Science
2	Data Collection	Collected bibliographic data from Web of Science.	Total Records: 8159 from nine distinct datasets.
3	Data Preparation	Preprocessing of text data for analysis.	Text cleaning, stop word removal, tokenization, and lemmatization.
4	Sentiment Analysis	Assessed sentiment of each document based on abstract content.	Sentiment categories: Positive, Negative, Neutral. Sentiment distribution visualized.
5	Topic Modeling	Applied LDA to uncover latent themes within the dataset.	Generated six topics, refined with coherence scoring and visualized.
6	Visualization	Displayed sentiment and topic distribution using graphs and word clouds.	Tools: WordCloud for keyword analysis, LDA visualization with pyLDAvis.
7	Interpretation and Conclusion	Provided insights on prominent research themes and sentiment trends.	Highlighted dominant topics and emerging research areas.

**Table 3:** Overview of topics, top words and average distributions.

Topic	Top Words	Average Distribution
1	smart, IoT, data, internet, systems	0.2001862
2	data, IoT, sensor, proposed, processing	0.12894942
3	data, big, IoT, healthcare, internet	0.15653505
4	IoT, computing, data, cloud, network, edge	0.1803717
5	data, IoT, security, model, proposed	0.17459249
6	industry, technologies, research, IoT	0.15936514

**Table 4:** Alpha and beta impact on topic distribution stability.

Topic	Average Distribution	Alpha	Beta
Topic 1	0.2462	0.1	0.15
Topic 2	0.1438	0.05	0.12
Topic 3	0.2466	0.1	0.2
Topic 4	0.1853	0.08	0.18
Topic 5	0.178	0.07	0.25
Topic 6	0.2142	0.9	0.22

**Table 5:** Key terms and their weights across topics.

Topic No.	Score 1	Score 2	Score 3	Score 4	Score 5	Score 6	Score 7	Score 8	Score 9	Score 10
0	data (0.011)	IoT (0.009)	technologies (0.009)	industry (0.008)	research (0.007)	smart (0.006)	digital (0.006)	systems (0.006)	paper (0.006)	system (0.005)
1	smart (0.012)	data (0.009)	IoT (0.007)	system (0.007)	paper (0.006)	model (0.005)	technology (0.005)	research (0.005)	industry (0.005)	information (0.004)
2	research (0.013)	industry (0.012)	technologies (0.010)	data (0.010)	study (0.006)	technology (0.006)	IoT (0.005)	computing (0.005)	supply (0.005)	paper (0.005)
3	data (0.023)	IoT (0.010)	system (0.007)	energy (0.007)	smart (0.007)	proposed (0.006)	paper (0.006)	big (0.006)	network (0.005)	devices (0.005)
4	data (0.022)	IoT (0.009)	learning (0.007)	proposed (0.006)	model (0.005)	results (0.005)	things (0.005)	internet (0.005)	devices (0.004)	based (0.004)
5	data (0.015)	model (0.013)	proposed (0.008)	system (0.008)	using (0.007)	energy (0.006)	based (0.006)	fiber (0.006)	results (0.006)	algorithm (0.005)

## Data Availability

The data will be made available upon request.
